# ﻿Three new species of Apseudomorpha (Crustacea, Tanaidacea) from Jiaozhou Bay, the Yellow Sea, and the South China Sea off coasts of China

**DOI:** 10.3897/zookeys.1096.79382

**Published:** 2022-04-15

**Authors:** You-Wei Tzeng, Lin Ma, Xinzheng Li

**Affiliations:** 1 Department of Marine Organism Taxonomy and Phylogeny, Institute of Oceanology, Chinese Academy of Sciences, Qingdao 266071, China Institute of Oceanology, Chinese Academy of Sciences Qingdao China; 2 University of Chinese Academy of Sciences, Beijing 100049, China University of Chinese Academy of Sciences Beijing China; 3 Center for Ocean Mega-Science, Chinese Academy of Sciences, Qingdao, 266071, China Center for Ocean Mega-Science, Chinese Academy of Sciences Qingdao China; 4 Laboratory for Marine Biology and Biotechnology, Pilot National Laboratory for Marine Science and Technology (Qingdao), Qingdao 266237, China Laboratory for Marine Biology and Biotechnology, Pilot National Laboratory for Marine Science and Technology (Qingdao) Qingdao China

**Keywords:** *Apseudes*, Apseudidae, Kalliapseudidae, Parapseudidae, *Phoxokalliapseudes*, *Swireapseudes*

## Abstract

Three new species of the crustacean order Tanaidacea are described from the coasts of China. *Apseudesspinidigitus***sp. nov.** (family Apseudidae), from the South China Sea, can be distinguished from the most similar species, *A.nhatrangensis*, by the features of the maxilliped endite, cheliped dactylus, pereopod 1 carpus and propodus, and pleopods basal article. *Phoxokalliapseudesshandongensis***sp. nov.** (family Kalliapseudidae), from Jiaozhou Bay and the Yellow Sea, can be differentiated from the most similar species, *P.gibbus*, by the features of the antennule article 1, pereopods 1 and 6 propodi, and uropod basal article. *Swireapseudesplanafrontis***sp. nov.** (family Parapseudidae), from Jiaozhou Bay, can be clearly separated from its congeners by its rostrum, antennule article 1, and pereopod 4 dactylus. A morphological key and comparison table of genus *Apseudes* from the South China Sea, as well as all known species of the genera *Phoxokalliapseudes* and *Swireapseudes* are provided.

## ﻿Introduction

**Figure 1.**
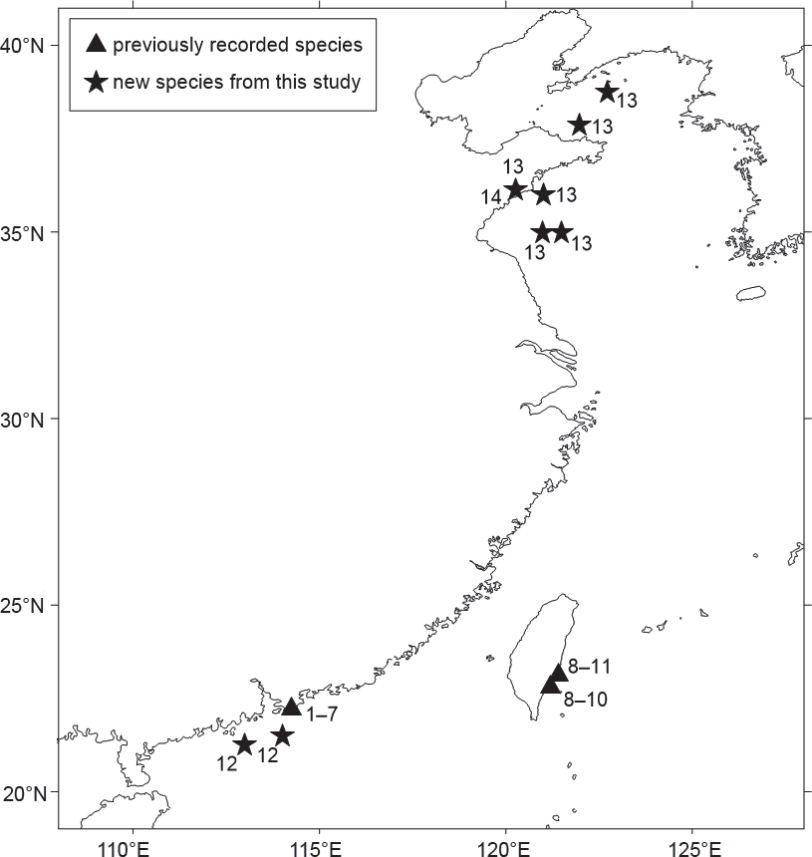
Distribution of Apseudomorpha from coasts of China: **1***Paradoxapseudesmortoni* (Bamber, 1997) **2**Discapseudes (Discapseudes) mackiei Bamber, 1997 **3***Swireapseudestoloensis* Bamber, 1997 **4***Siegiusgallardoi* (Shiino, 1963) **5***Unguispinosushodgsoni* (Bamber, 2000) **6***Tanapseudessinensis* Bamber, 2000 **7***Apseudesmanna* Bamber, 2008 **8***Paradoxapseudespangcahi* Tzeng & Hsueh, 2014 **9***Pseudoapseudomorphatagopilosus* Tzeng & Hsueh, 2014 **10***Synapseudeshansmuelleri* Guţu, 2006 **11***Indoapseudesmultituberculata* Tzeng & Hsueh, 2014 **12***Apseudesspinidigitus* sp. nov. **13***Phoxokalliapseudesshandongensis* sp. nov. **14***Swireapseudesplanafrontis* sp. nov.

The suborder Apseudomorpha Sieg, 1980 contains 12 families, including more than 530 extant species recorded to date (WoRMS 2021). All the species of the suborder are small-sized crustaceans mainly below 15 mm that widely inhabit various shallow marine benthic habitats, especially abundant in coral reefs, estuaries, and mangroves from the tropics to temperate waters (Błażewicz-Paszkowycz et al. 2012). Despite their wide distribution in shallow waters, knowledge of species diversity and composition from many areas is still lacking. For example, studies on taxonomy of apseudomorph tanaidaceans from the coasts of China were only intensively focused on the adjacent waters of Hong Kong (Fig. 1; Bamber 1997, 2000, 2008; Bamber and Sheader 2003) and eastern Taiwan (Fig. 1; Tzeng and Hsueh 2014). There is a knowledge gap of more than a decade, and species from the extensive coastline of China remain unknown.

In the present study, three species of Apseudomorpha from coasts of China are recognized. The first species has a well separated eye lobe, one spine-like anterolateral apophysis on pereonites 3–6, denticulate outer margin of mandible, fossorial type of pereopod 1, and one spine-like apophysis on pereopod 1 coxa, belonging to *Apseudes* Leach, 1814, a worldwide genus with over 40 species (Larsen et al. 2011). The second species has ventral spiniform setae on antennule peduncle article 1, numerous long plumose setae on mandible palp, maxilliped palp and cheliped, one distal spiniform seta on mandible palp, four cusps on labrum, very long male pereopod 6 dactylus, and two posterior plumose setae on pleotelson, fitting the diagnosis of *Phoxokalliapseudes* Drumm & Heard, 2011, a genus with species all recorded from western Pacific by far (Drumm and Heard 2011). The third species has fossorial type of pereopod 1 but without spine-like apophysis on coxa, chelate or subchelate dactylus on all pereopods, long and thin pereopod 6 basis, conforming to the features of *Swireapseudes* Bamber, 1997, a small genus with only two species, recorded from the South China Sea and Northwestern Atlantic Ocean (Guţu 2008). Morphological examination confirms that all three species are different from their congeners and are recognised as new to science, and they are described here.

## ﻿Materials and methods

The specimens of Apseudomorpha were collected from Jiaozhou Bay, the Yellow Sea, and the South China Sea, from 3 to 55 m depth. The first species, belonging to the genus *Apseudes*, were collected from the South China Sea off Guangdong Province during the National Comprehensive Oceanography Survey cruise carried out in 1959–1964. The second species, belonging to the genus *Phoxokalliapseudes*, was collected during the Jiaozhou Bay cruise carried out in 1959–1964, the quarterly Jiaozhou Bay survey cruise during 2003–2021, and the Yellow Sea cruise conducted in 2019. The third species, belongs to the genus *Swireapseudes*, was also collected during the quarterly Jiaozhou Bay survey cruise. The collecting sites of new species from this study, as well as the distribution of previously recorded species from coasts of China, are shown in Fig. 1. All samples were collected using a box corer or a grab, then sieved over a 0.5 mm mesh and specimens were preserved in 75% alcohol or 5% neutral formalin. The specimens were examined and dissected under a stereo microscope (Zeiss Stemi 2000-C). Body parts and appendages of the specimens were examined and photographed using a Nikon Eclipse Ni. Digital images were enhanced with the computer software Helicon Focus 7.0.2. Drawings were prepared by tracing outlines of examined body parts and appendages from digitised images using CorelDRAW 2020. The descriptions of species are mostly based on paratypes and allotypes. All the measurements and proportions of the morphological structures were based on maximum width, except body length which was measured from rostrum tip to the pleotelson apex. Terminology used in the present study mainly follows Drumm and Heard (2011). All specimens of this study were deposited at the Marine Biological Museum, Chinese Academy of Sciences (**MBM**, or **MBMCAS**), in the Institute of Oceanology, Chinese Academy of Sciences, Qingdao, China.

## ﻿Systematics

**Order Tanaidacea Dana, 1849**

**Suborder Apseudomorpha Sieg, 1980**

**Superfamily Apseudoidea Leach, 1814**

### Family Apseudidae Leach, 1814

**Subfamily Apseudinae Leach, 1814**

#### ﻿Genus Apseudes

Taxon classificationAnimaliaTanaidaceaApseudidae

Leach, 1814

03C8C651-94DF-5AE4-92CE-B12D767BC2BE

##### Type species.

*Apseudestalpa* (Montagu, 1808)

#### ﻿ Apseudes spinidigitus sp. nov.

Taxon classificationAnimaliaTanaidaceaApseudidae

2BF55C22-9811-5B29-B286-B1B782359868

http://zoobank.org/025453A4-9F04-4F9F-8FD3-C16CB633FAAF

**Figure 2.**
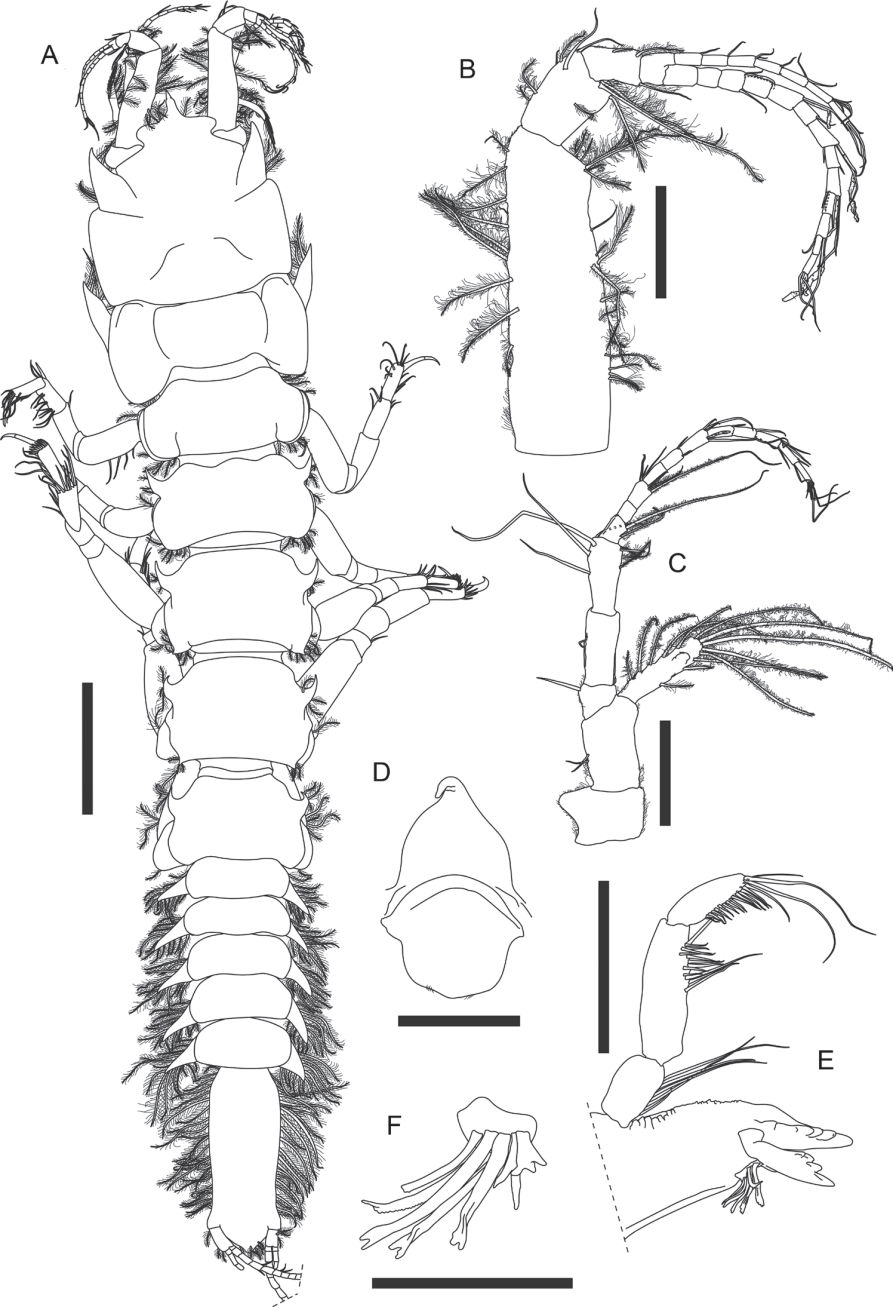
*Apseudesspinidigitus* sp. nov. Paratype (MBM032095), non-ovigerous simultaneous hermaphrodite**A** body dorsal view **B** right antennule **C** right antenna **D** epistome and labrum **E** left mandible **F** right mandible setal row. Scale bars: 1 mm (**A**); 0.2 mm (**B, C, E**); 0.1mm (**D, F**).

**Figure 3.**
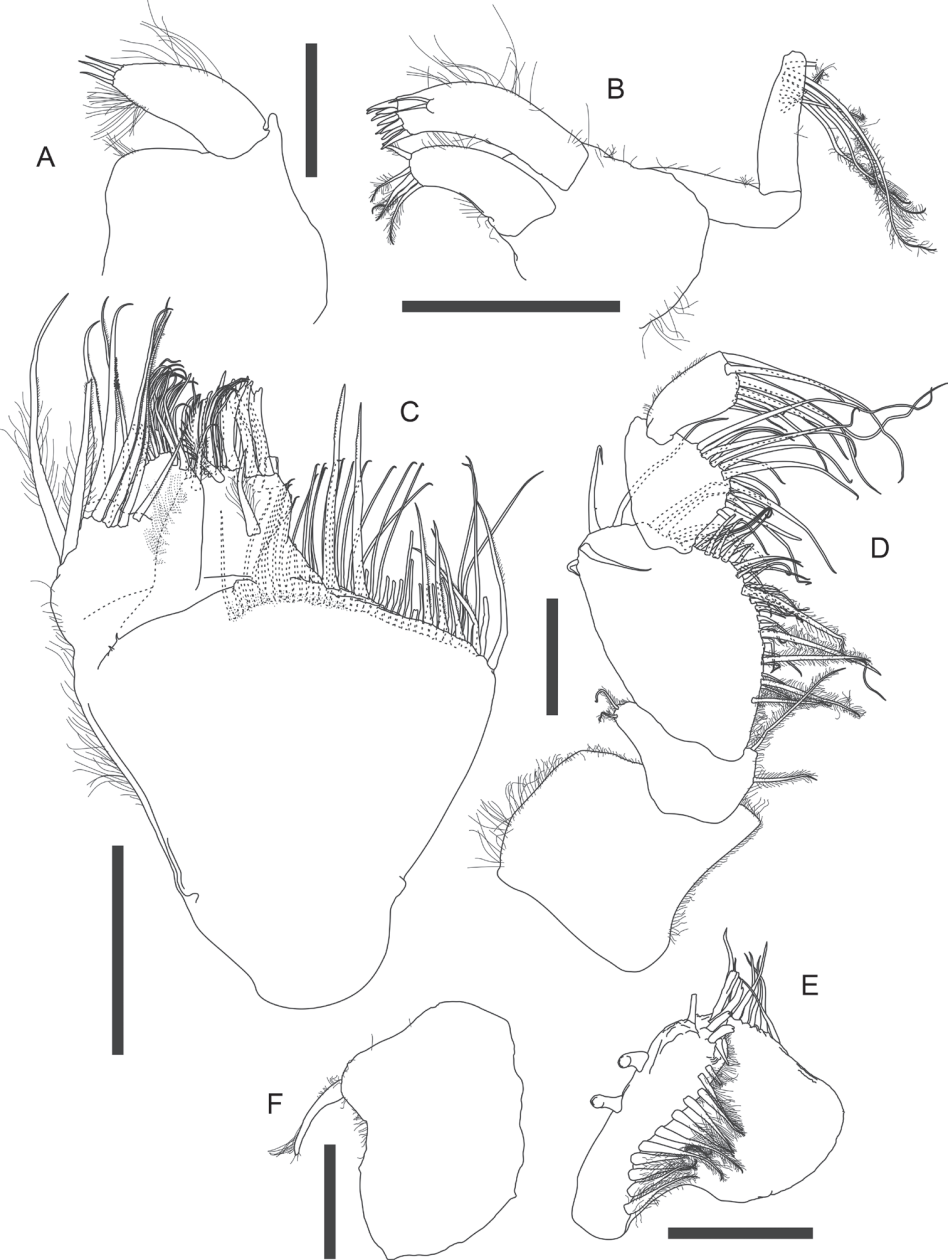
*Apseudesspinidigitus* sp. nov. Paratype (MBM032095), non-ovigerous simultaneous hermaphrodite**A** labium **B** maxillule **C** maxilla **D** maxilliped **E** maxilliped endite **F** epignath. Scale bars: 0.1 mm (**A, D–F**); 0.2 mm (**B, C**).

**Figure 4.**
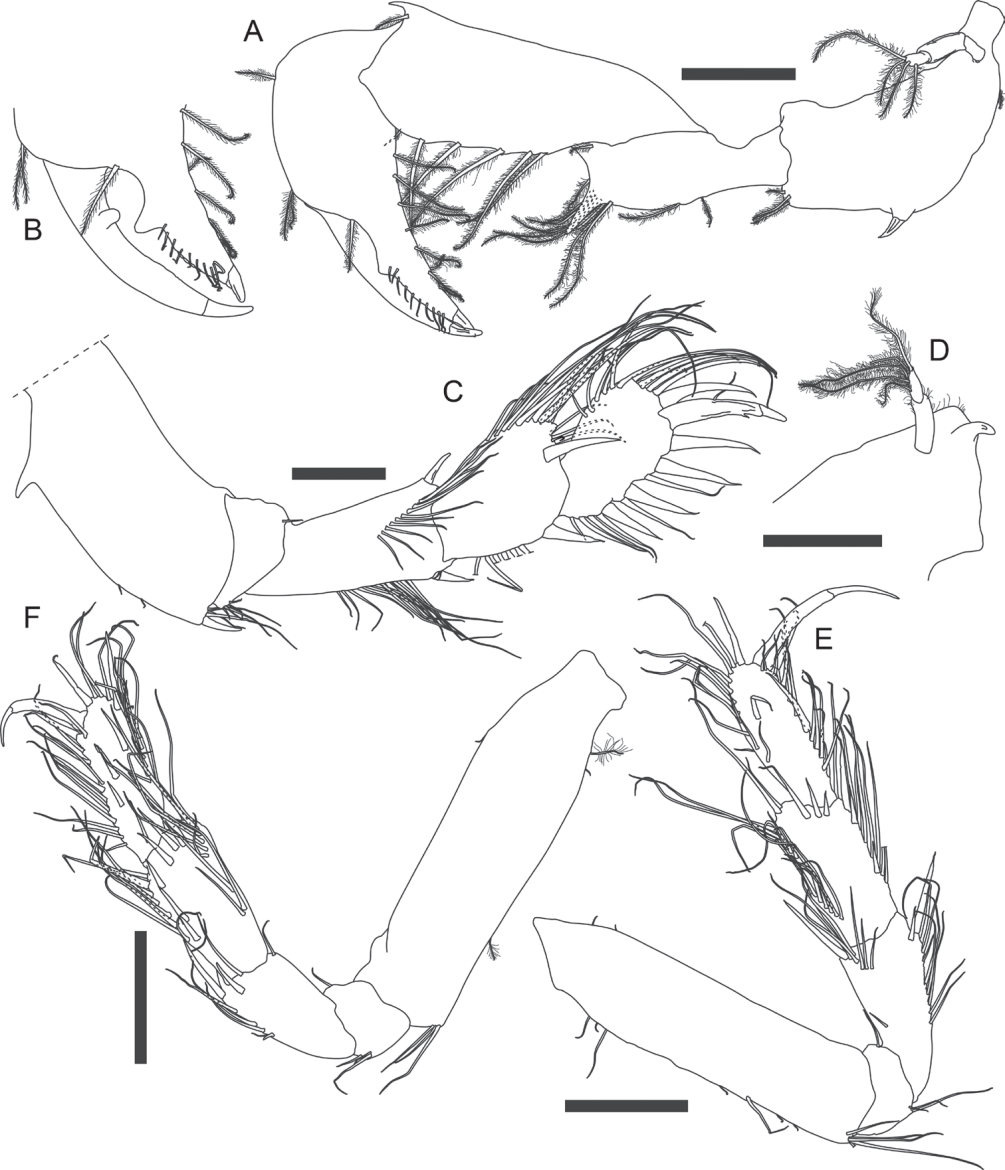
*Apseudesspinidigitus* sp. nov. Paratype (MBM032095), non-ovigerous simultaneous hermaphrodite**A** left cheliped **B** left cheliped fixed finger and dactylus **C** right pereopod 1 **D** proximal end of left pereopod 1 basis **E** right pereopod 2 **F** left pereopod 3. Scale bars: 0.2 mm.

**Figure 5.**
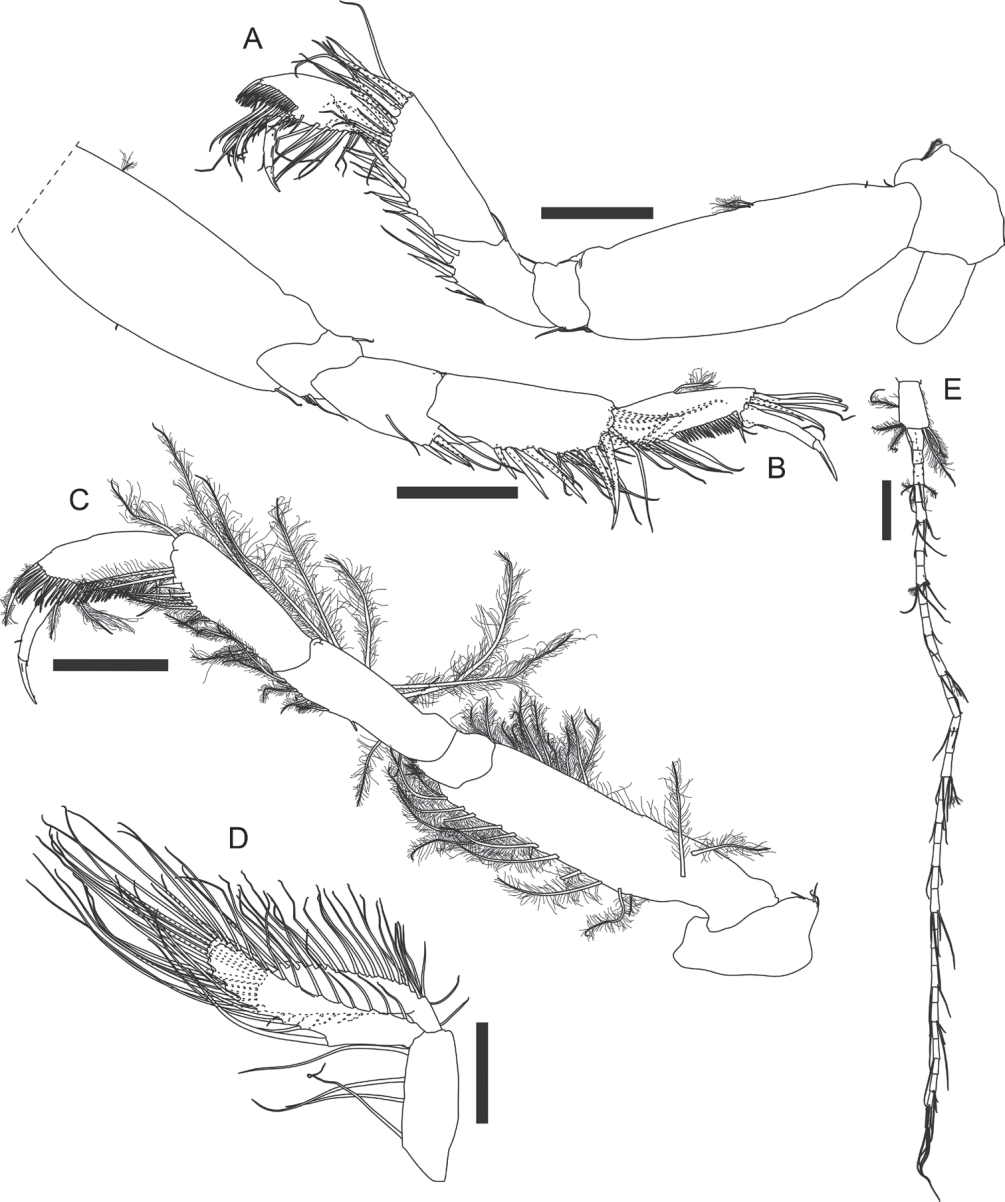
*Apseudesspinidigitus* sp. nov. Paratype (MBM032095), non-ovigerous simultaneous hermaphrodite**A** left pereopod 4 **B** right pereopod 5 **C** left pereopod 6 **D** left pleopod 5 (all setules omitted) **E** left uropod. Scale bars: 0.2 mm.

Figs 2 , 3 , 4 , 5 , Table 1

##### Type material.

***Holotype***: MBM287293, non-ovigerous simultaneous hermaphrodite, 8.3 mm; the South China Sea off Guangdong Province, China, 21 October 1959, from mud-sandy substrate at a depth of 43 m, 21°15'N, 113°00'E. ***Paratypes***: MBM032095, one non-ovigerous simultaneous hermaphrodite, 8.5 mm, completely dissected and body parts preserved in 75% alcohol; the South China Sea off Guangdong Province, China, 10 January 1960, from muddy substrate at a depth of 55 m, 21°30'N, 114°00'E. MBM032282, four non-ovigerous simultaneous hermaphrodites; same collection data as holotype.

##### Type locality.

Northern South China Sea.

##### Etymology.

The name is derived from the Latin *spinosus* (spinous) and *digitus* (finger), referring to the dactylus and fixed finger of cheliped both equipped with one conspicuous apophysis on the incisive margins.

##### Diagnosis.

***Non-ovigerous simultaneous hermaphrodite. Rostrum*** cordiform, distally pointed. ***Carapace*** lateral margin with one large spine-like anterior apophysis. ***Pereonites****3–6* with one curved spine-like anterolateral apophysis. ***Maxilliped*** endite inner margin with two coupling hooks. ***Cheliped*** fixed finger and dactylus incisive margin with one conspicuous apophysis, respectively. ***Pereopod 1*** merus and carpus each with one dorsodistal and one ventrodistal spiniform seta; propodus with one dorsodistal and four ventral spiniform setae. ***Pleopod*** basal article with four inner plumose setae.

##### Description.

***Simultaneoushermaphrodite* (non-ovigerous paratype MBM032095). *Body*** (Fig. 2A) dorsoventrally flattened, 8.5 mm long, 5.8× as long as broad, posteriorly narrower. ***Carapace*** subrectangular, ~ 0.2× as long as total body length, 1.1× as long as broad; rostrum cordiform, with proximal half laterally extended, distal half narrow, terminally pointed and slightly down-curved; lateral margin with one large spine-like anterior apophysis adjacent to eye lobe, with three outer and three inner plumose setae on apophysis. ***Eye lobe*** well separated, wide and short, without visual elements. ***Pereon*** ~ 0.5× as long as total body length; pereonite 1 broadest, slightly broader than carapace, 0.5× as long as broad; pereonite 2 0.7× as long as pereonite 1, 0.5× as long as broad, with *ca.* three anterolateral and five posterolateral plumose setae; pereonite 3 slightly longer than pereonite 2, 0.5× as long as broad, anterolateral margin with one curved spine-like apophysis and three plumose setae, posterolateral margin with *ca.* seven plumose setae; pereonite 4 1.2× as long as pereonite 3, 0.7× as long as broad, anterolateral margin with one curved spine-like apophysis and three plumose setae, posterolateral corner pointed, with *ca.* seven plumose setae; pereonite 5 similar to pereonite 4 but with three posterolateral plumose setae; pereonite 6 0.9× as long as pereonite 5, 0.7× as long as broad, anterolateral margin with one curved spine-like apophysis and *ca.* three plumose setae, pereonites 1–5 each with one hyposphenia, pereonite 6 with genital cone. ***Pleon*** as long as carapace, posteriorly narrower, each pleonite with pointed epimera and ~ 12 lateral plumose setae. ***Pleotelson*** 0.9× as long as carapace, 2.5× as long as broad, lateral margin with > 20 plumose setae, terminally subtriangular, with *ca.* six posterior plumose setae.

***Antennule*** (Fig. 2B) peduncle article 1 3.2× as long as broad, outer margin with ~ 17 circumplumose setae, two broom setae and one simple seta, inner margin with nine circumplumose setae; article 2 short, 0.3× as long as article 1, 1.8× as long as broad, with *ca.* seven circumplumose setae and three simple setae; article 3 0.6× as long as article 2, 1.7× as long as broad, with three circumplumose setae; article 4 naked, 0.5× as long as article 3, *ca.* as long as broad; outer flagellum 13-articled, articles 3, 5, 7, 9, 11, and 13 with one distal aesthetasc and 1–4 distal simple setae, other articles with one distal simple seta or naked; inner flagellum 7-articled, articles 1–5 with one or two distal simple setae, articles 6 and 7 with four distal simple setae.

***Antenna*** (Fig. 2C) peduncle article 1 short and covered with setules, 0.9× as long as broad, inner margin with conical apophysis; article 2 1.6× as long as article 1, 1.7× as long as broad, with two inner simple setae; squama slender, slightly longer than article 2, 3.8× as long as broad, with 13 circumplumose setae; article 3 short, 0.4× as long as article 2, 0.9× as long as broad, with one inner distal simple seta; article 4 0.8× as long as article 2, 2.4× as long as broad, with one inner simple seta; article 5 0.8× as long as article 2, 2.2× as long as broad, outer margin with three circumplumose and one broom seta, inner margin with four long simple setae; flagellum 11-articled, article 1 with two long distal circumplumose setae, article 3 with two long distal circumplumose setae and five distal simple setae, article 6 with one distal aesthetasc and four distal simple setae, article 11 with eight distal simple setae, and other articles with 1–4 simple setae or naked.

***Epistome*** (Fig. 2D) with one strong and curved apical apophysis. *Labrum* (Fig. 2D) rounded with some setules.

***Left mandible*** (Fig. 2E) outer margin denticulate; incisor with four or five denticles; lacinia mobilis large and subrectangular, distal margin with four denticles; setal row with four serrate setae and three simple setae; molar not examined; palp 3-articled, article 1 1.7× as long as broad, inner margin with five simple setae, article 2 shorter than articles 1 and 3 combined, 3× as long as broad, inner margin with 12 simple setae and one very long simple seta, article 3 1.2× as long as article 1, 3× as long as broad, inner margin with 15 simple setae and two very long simple setae. *Right mandible* similar to left mandible but without lacinia mobilis, setal row (Fig. 2F) with one trifurcate, four bifurcate, one serrate, and one blunt seta.

***Labium*** (Fig. 3A) antero-outer corner near palp with one small apophysis; palp large and covered with setules, with three distal simple setae.

***Maxillule*** (Fig. 3B) covered with setules; inner endite with five distal plumose setae; outer endite with two subdistal simple setae and ten distal spiniform setae; palp 2-articled, article 2 with seven subdistal plumose setae.

***Maxilla*** (Fig. 3C) outer margin covered with setules; outer lobe of movable endite with two plumose setae and seven serrate setae; inner lobe of movable endite with a row of six blunt setae, a cluster of > 13 simple setae, and two serrate setae; outer lobe of fixed endite with one comb-like seta, two trifurcate setae, one bifurcate seta, two plumose setae, and > 16 simple setae, outer margin covered with setules; inner lobe of fixed endite with two long setae only serrate on distal 1/4, five serrate setae, and ~ 39 simple setae along distal margin.

***Maxilliped*** (Fig. 3D, E) basis covered with setules; endite (Fig. 3E) inner margin with two coupling hooks, inner fold with 11 circumplumose setae, distal margin with two blunt, three bifurcate, and eight simple setae; palp 4-articled, article 1 with two short outer-distal circumplumose setae and two inner circumplumose setae, article 2 outer-distal, distal, and inner margin with > 37 simple and five circumplumose setae; article 3 inner margin with ~ 16 simple setae; article 4 distal margin with nine simple setae.

***Epignath*** (Fig. 3F) typical of genus, partially covered with setules, with one stout plumose seta.

***Cheliped*** (Fig. 4A) exopod 3-articled, article 3 with four plumose setae; basis 2.2× as long as broad, ventral margin with one proximal plumose seta, one strong spiniform seta midway, and three distal plumose setae; merus 0.8× as long as basis, 3× as long as broad, ventral margin with two plumose setae midway, one very large subdistal spine-like apophysis, eight subdistal and one distal plumose seta; carpus elongate, 1.2× as long as basis, 2.8× as long as broad, with one dorsodistal spine-like apophysis, one dorsodistal plumose seta, and six plumose setae along ventral margin; propodus palm 1.4× as long as broad, with one dorsoproximal plumose seta, two dorsodistal plumose setae, one plumose setae near dactylus articulation, and eight plumose setae along ventral margin of palm and fixed finger; fixed finger (Fig. 4B) nearly as long as palm, ~ 2.1× as long as broad, incisive margin with one large apophysis and ten simple setae on distal half; dactylus (Fig. 4B) plus unguis 4.3× as long as broad, slightly curved, incisive margin with one conspicuous apophysis.

***Pereopod 1*** (Fig. 4C, D) coxa (Fig. 2A) with large and pointed spine-like apophysis and five plumose setae; exopod 2-articled, article 2 with five plumose setae; basis ~ 2.4× as long as broad, with one subdorsal proximal apophysis near exopod, ventral margin with one subproximal apophysis, two subdistal simple setae, four distal simple setae and one distal spiniform seta; ischium with one short dorsodistal simple seta and four short ventrodistal simple setae; merus 2.2× as long as broad, with one dorsodistal spiniform seta and a row of ~ 11 lateral simple setae, ventral margin with a row of ~ 11 simple setae on distal half, one distal spiniform seta and two short distal simple setae; carpus short and thick, dorsally extended, 0.7× as long as merus, 1.3× as long as broad, dorsal margin with a row of ~ 12 long simple setae on distal half, one short and three longer distal simple seta, and one large distal spiniform seta, ventral margin with six simple setae midway and one large distal spiniform seta; propodus *ca.* as long as carpus, slightly thinner than carpus, dorsal margin with ~ 11 simple setae on distal half, and one large distal spiniform seta, ventral margin with six simple setae and four large spiniform setae; dactylus plus unguis 0.9× as long as propodus, slightly curved, dorsal margin with one simple seta midway, ventral margin with one subdistal spinule.

***Pereopod 2*** (Fig. 4E) coxa with one plumose seta; basis 4× as long as broad, dorsal margin with two short simple setae on proximal half, ventral margin with one short and two longer simple setae on proximal half, three short and one longer simple seta on distal half, and a cluster of five distal simple setae; ischium with one dorsodistal and three ventrodistal simple setae; merus ~ 0.5× as long as basis, 2.3× as long as broad, with one subdorsal simple seta on proximal half, a row of six dorsodistal simple setae, one long and slender dorsodistal spiniform seta, ventral margin with eight simple setae along distal half, one long slender subdistal spiniform seta and one shorter subdistal spiniform seta; carpus 0.8× as long as merus, 2× as long as broad, dorsal margin with a row of *ca.* seven simple setae on proximal half, *ca.* six simple setae on distal half, and two distal simple setae, distal margin with a row of three lateral spiniform setae, ventral margin with eight simple setae, one long and slender spiniform seta, and two shorter spiniform setae; propodus slightly longer than carpus, 2.9× as long as broad, with ~ 12 simple setae along distal half of dorsal margin, one long and slender dorsodistal spiniform seta, and one lateral spiniform seta, ventral margin with ~ 11 simple setae, one long and slender spiniform seta midway and one distal spiniform seta; dactylus plus unguis thin and curved, nearly as long as propodus, with one doral seta midway, unguis ~ 0.7× as long as dactylus.

***Pereopod 3*** (Fig. 4F) basis 3.7× as long as broad, dorsal margin with one short simple seta on proximal half, ventral margin with one broom and one short simple seta on proximal half, one broom seta on distal half, and three simple distal setae; ischium with one dorsodistal and three ventrodistal simple setae; merus 0.4× as long as basis, 2.2× as long as broad, with one dorsodistal simple seta, one distolateral simple seta, and one distolateral spiniform seta, ventral margin with four simple setae on distal half, one small spiniform seta midway, and one long and slender distal spiniform seta; carpus slightly longer than merus, 2.1× as long as broad, with three long simple setae on proximal half, a row of *ca.* seven simple setae on distal half of dorsal margin, and a row of six lateral spiniform setae, distal one longer, ventral margin with six simple setae and one spiniform seta along distal half; propodus slightly longer than carpus, 2.8× as long as broad, with ~ 11 simple setae and two long and slender spiniform setae on distal half of dorsal margin, three longer and one very short lateral spiniform seta, ventral margin with 11 simple setae and one distal spiniform seta; dactylus plus unguis thin and curved, ~ 0.7× as long as propodus, unguis 0.5× as long as dactylus.

***Pereopod 4*** (Fig. 5A) coxa with one plumose seta; basis 2.9× as long as broad, dorsal margin with two short proximal simple setae and two broom setae midway, ventral margin with two longer and one short distal simple seta; ischium with one dorsodistal simple seta and one ventrodistal simple seta; merus ~ 0.4× as long as basis, 2.3× as long as broad, dorsal margin with one distal simple seta, ventral margin with three simple setae midway, four spiniform setae and two simple setae on distal half; carpus ~ 1.2× as long as merus, 2.7× as long as broad, with ~ 11 spiniform setae and > 17 simple setae along ventral to distal margin; propodus shorter and thinner than carpus, 0.8× as long as carpus, 2.9× as long as broad, with a comb-like row of numerous short and fine dorsodistal serrate setae and a cluster of numerous longer dorsodistal simple setae, ventral margin with ~ 11 longer simple setae and *ca.* seven short simple setae; dactylus plus unguis slender and slightly curved, 0.6× as long as propodus, dorsal margin with one seta midway, unguis 0.5× as long as dactylus.

***Pereopod 5*** (Fig. 5B) basis dorsal margin with one broom seta on proximal half, ventral margin with one short midway and one longer distal simple seta; ischium with one dorsal and three ventrodistal simple setae; merus 2.4× as long as broad, with one dorsodistal, one subventral, two ventrodistal simple setae, and three ventrodistal spiniform setae; carpus slightly longer and thinner than merus, 2.6× as long as broad, with *ca.* ten spiniform setae and > 19 simple setae along ventral to distal margin; propodus slender, 0.8× as long as carpus, 3.2× as long as broad, dorsal margin with one broom seta midway and five strong distal simple setae, ventral margin with seven simple setae on proximal half, two distal simple setae, one strong spiniform seta midway, one distal spiniform seta, and a comb-like row of ~ 20 short and fine serrate setae between those two spiniform setae; dactylus plus unguis slender and slightly curved, 0.8× as long as propodus, dorsal margin with one simple seta midway, unguis 0.6× as long as dactylus.

***Pereopod 6*** (Fig. 5C) coxa with three simple setae; basis 4× as long as broad, with one lateral and one subdorsal circumplumose seta on proximal half, dorsal margin with 11 circumplumose setae along distal half, ventral margin with one small spiniform seta midway and 12 circumplumose setae; ischium with three ventrodistal circumplumose setae; merus 0.6× as long as basis, 3.1× as long as broad, dorsal margin with one shorter and two very long circumplumose setae on proximal half, three very long circumplumose setae on distal half, ventral margin with two circumplumose setae on proximal half, one small spiniform seta midway, five circumplumose setae and three small spiniform setae on distal half; carpus slightly shorter than merus, 2.9× as long as broad, dorsal margin with two midway and one distal very long circumplumose seta, ventral to distal margin with ~ 13 circumplumose setae and one distal spiniform seta; propodus 0.8× as long as carpus, 2.9× as long as broad, with two ventroproximal circumplumose setae, a comb-like row of numerous serrate setae along ventral to distal margin, one dorsodistal and two ventrodistal circumplumose setae; dactylus plus unguis slender and slightly curved, 0.9× as long as propodus, dorsal margin with one short simple seta midway, unguis 0.6× as long as dactylus.

***Pleopods*** (Fig. 5D, all setae plumose, setules omitted in figure) basal article elongate, 2.7× as long as broad, with four inner plumose setae; exopod slender, 3.6× as long as broad, outer to distal margin with 33 plumose setae, inner margin with seven short simple setae; endopod longer and slender than exopod, 1.2× as long as exopod, 5× as long as broad, with 25 plumose setae and one stronger plumose seta on proximal half of inner margin.

***Uropod*** (Fig. 5E) basal article 1.6× as long as broad, outer margin with one midway and three distal circumplumose setae, inner margin with two distal setae; exopod not examined; endopod 36-articled, articles 3, 8, 13, and 18 with 1–3 broom setae and 1–5 simple distal setae, other articles without seta or with at most six simple setae.

##### Variation.

Non-ovigerous simultaneous hermaphrodite holotype (MBM287293, 8.3 mm long) uropod with 8-articled exopod and 38-articled endopod.

##### Remarks.

According to Bamber (1998, 2008), only three species of the genus *Apseudes* have been recorded from South China Sea region previously: *A.manna* Bamber, 2008 (Hong Kong), *A.nagae* Shiino, 1963 (Vietnam, Sabah and Brunei), and *A.nhatrangensis* Shiino, 1963 (Vietnam). Among these three, *A.manna* is the geographically closest to *Apseudesspinidigitus* sp. nov.; the new species, however, is conspicuously distinguished from *A.manna* in morphology, by having the spine-like apophyses on carapace, pereonites, merus, carpus, and fingers of cheliped, and pointed epimera on each pleonite (Fig. 2A, 4A, B, Table 1; Bamber 2008: figs 1A, 2A).

**Table 1.** Morphological comparison among species of the genus *Apseudes* from the South China Sea region. N = no apophyses or spiniform setae.

Character / Species name	*A.spinidigitus* sp. nov.	*A.manna*	*A.nagae*	*A.nhatrangensis*
**Carapace**				
Rostrum	cordiform, pointed	pointed	cordiform, pointed	cordiform, pointed
Lateral apophyses	one spine-like	N	N	one spine-like
**Pereonites**				
Pereonite 3–6 lateral apophyses	one curved spine-like	N	blunt	one curved spine-like
**Pleonites**				
Epimera	pointed	blunt	truncated	pointed
**Antennule**				
Number of outer/inner flagellum articles	13/7	10/5	10–14/5	13/9
**Antenna**				
Number of flagellum articles	11	8	8	10
**Maxilliped**				
Number of endite coupling hooks	2	2	3–4	4
**Cheliped**				
Basis apophyses or spiniform setae	one ventral spiniform seta	one ventral spiniform seta	one ventral spiniform seta	one large ventral apophysis
Merus apophyses or spiniform setae	one ventral subdistal apophysis	N	N	one ventral subdistal apophysis
Carpus apophyses or spiniform setae	one dorsodistal apophysis	N	N	N
**Pereopod 1**				
Number of basis/merus/carpus/propodus dorsal spiniform setae	0/1/1/1	0/1/1/2	0/1/1/2	0/1/2/2
Number of basis/merus/carpus/propodus ventral spiniform setae	1/1/1/4	3/1/2/5	1/1/2/4	1/0/0/4
**Pleopods**				
Number of basal article outer/inner setae	0/4	5/4	4/5	8/8
**Uropod**				
Number of exopod/endopod articles	8/36–38	4–5/multi	13/26	10/40
**References**	present study	Bamber 2008	Shiino 1963; Bamber 1998	Shiino 1963

*Apseudesspinidigitus* also closely resembles *A.nhatrangensis* in morphology. Among all known species of *Apseudes*, only *A.nhatrangensis* and the new species have the combination of features of a cordiform and distally pointed rostrum, one large spine-like anterior apophysis on lateral margin of carapace, a pair of wide but short eye lobes without visual elements, one large and curved spine-like anterolateral apophysis on pereonites 3–6, similar numbers of antennule and antenna flagella, uropod exopod and endopod articles, one large spine-like subdistal apophysis on ventral margin of cheliped merus, and one subproximal apophysis on ventral margin of pereopod 1 basis. Nevertheless, there are still several differences between these two species: 1) having two coupling hooks on maxilliped endite in *A.spinidigitus*, vs. four in *A.nhatrangensis*, 2) having a distinct spiniform seta midway on ventral margin of cheliped basis in *A.spinidigitus*, vs. a large apophysis in *A.nhatrangensis*, 3) the presence of one dorsodistal spine-like apophysis on cheliped carpus in *A.spinidigitus*, absence in *A.nhatrangensis*, 4) the presence of one conspicuous apophysis on incisive margin of cheliped dactylus in *A.spinidigitus*, vs. absence in *A.nhatrangensis*, 5) the presence of one ventrodistal spiniform seta on pereopod 1 merus and carpus each in *A.spinidigitus*, vs. absence in *A.nhatrangensis*, 6) having only one dorsodistal spiniform seta on pereopod 1 carpus and propodus each in *A.spinidigitus*, vs. two in *A.nhatrangensis*, 7) having only four plumose setae on inner margin of pleopods basal articles in *A.spinidigitus*, vs. more than four plumose setae on both sides of pleopods basal articles in *A.nhatrangensis* (Figs 2A–C, 3E, 4A–C, 5D, E, Table 1; Shiino 1963: figs 1A, C, D, 2A, H, I, 3A, B, D, E, G, H, 3H).

In addition to the features mentioned above, the presence of circumplumose setae on the antennule, antenna, maxilliped, pereopod 6 and uropod of *A.spinidigitus* is also a rare feature only found in a few species among *Apseudes*, i.e., *A.fecunda* (Guţu, 2006), *A.nagae*, *A.nhatrangensis*, *A.nipponicus* Shiino, 1937, *A.sculptus* Pfeffer, 1888 (Lang 1953), and *A.spectabilis* Studer, 1884 (Shiino 1978).

#### ﻿Key to the species of *Apseudes* from the South China Sea (see also Table 1)

**Table:** 

1	All pereonites without apophysis	***A.manna***
–	Pereonites 3–6 with one anterolateral apophysis	**2**
2	Carapace without lateral apophysis	***A.nagae***
–	Carapace with one large spine-like anterolateral apophysis	**3**
3	Cheliped basis with one large apophysis on ventral margin, dactylus incisive margin without apophysis	***A.nhatrangensis***
–	Cheliped basis with one large spiniform seta on ventral margin, dactylus incisive margin with one apophysis	***A.spinidigitus* sp. nov.**

### Family Kalliapseudidae Lang, 1956

**Subfamily Kalliapseudinae Lang, 1956**

#### ﻿Genus Phoxokalliapseudes

Taxon classificationAnimaliaTanaidaceaKalliapseudidae

Drumm & Heard, 2011

EDA1433B-B838-5CE3-B4CB-FDB388167618

##### Type species.

*Kalliapseudesgobinae* Bamber, 1998.

#### ﻿ Phoxokalliapseudes shandongensis sp. nov.

Taxon classificationAnimaliaTanaidaceaKalliapseudidae

BB28D5B7-6BCE-55C3-9A11-8673106C3429

http://zoobank.org/C248280D-BEC1-4FDC-819B-926AF126929D

**Figure 6.**
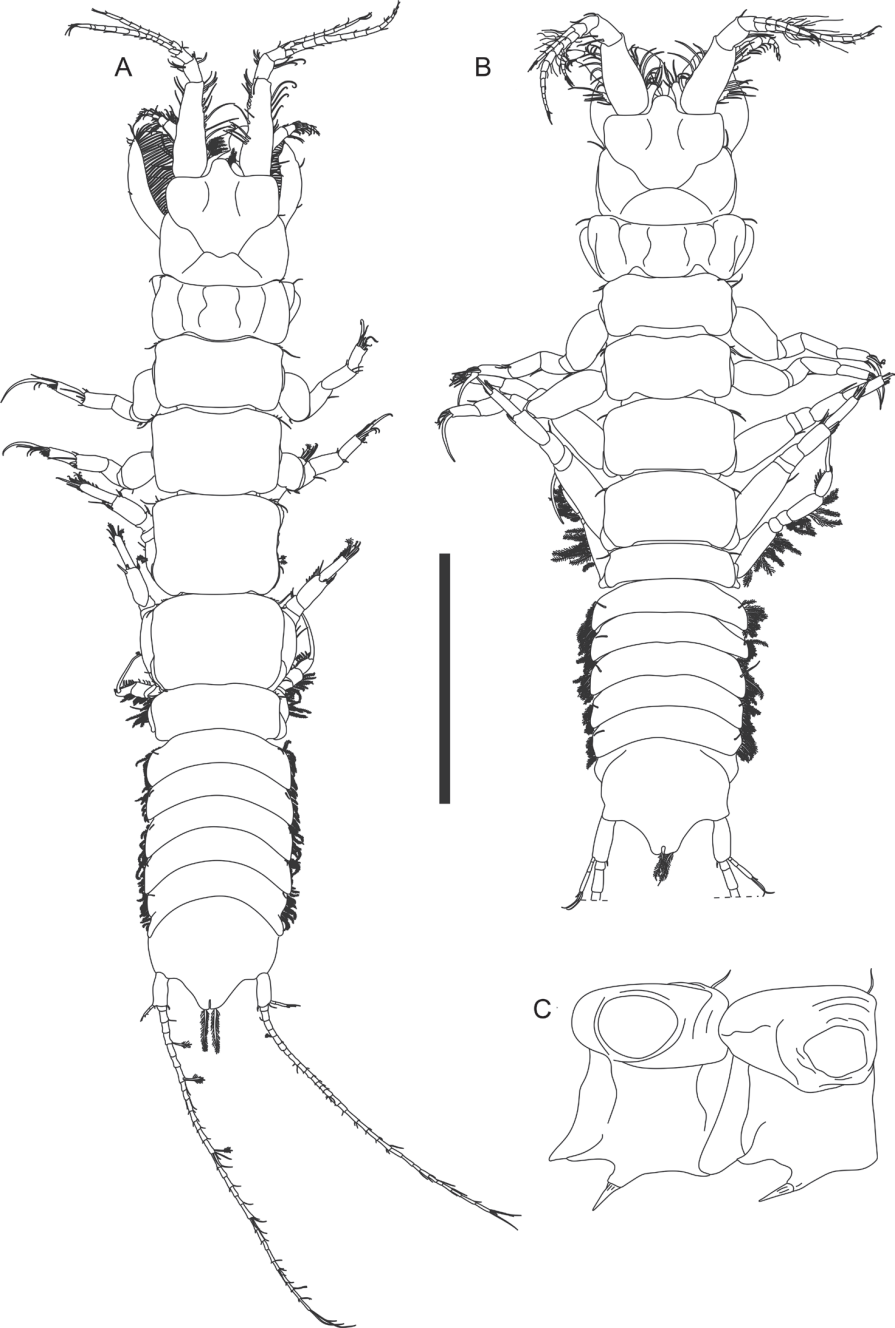
*Phoxokalliapseudesshandongensis* sp. nov. Paratype (MBM287294), non-ovigerous female **A** body dorsal view. Allotype (MBM287295), male **B** body dorsal view **C** pereonites 1–2 lateral view. Scale bar: 2 mm.

**Figure 7.**
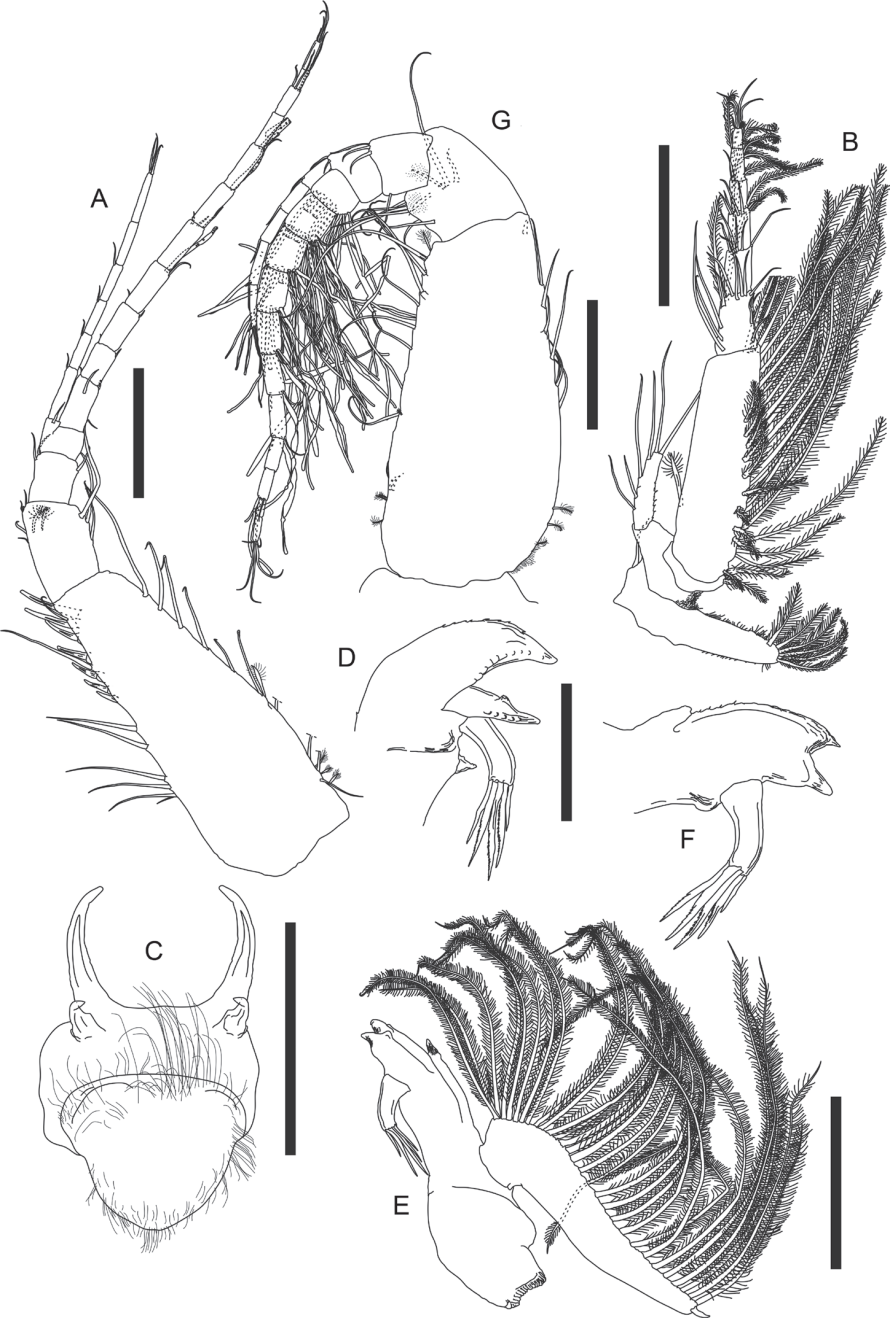
*Phoxokalliapseudesshandongensis* sp. nov. Paratype (MBM287294), non-ovigerous female **A** right antennule **B** left antenna **E** right mandible. Paratype (MBM287296), non-ovigerous female **C** labrum **D** left mandible incisor, lacinia mobilis and setal row **F** right mandible incisor and setal row. Allotype (MBM287295), male **G** left antennule. Scale bars: 0.2 mm (**A–C, E, G**); 0.1 mm (**D, F**).

**Figure 8.**
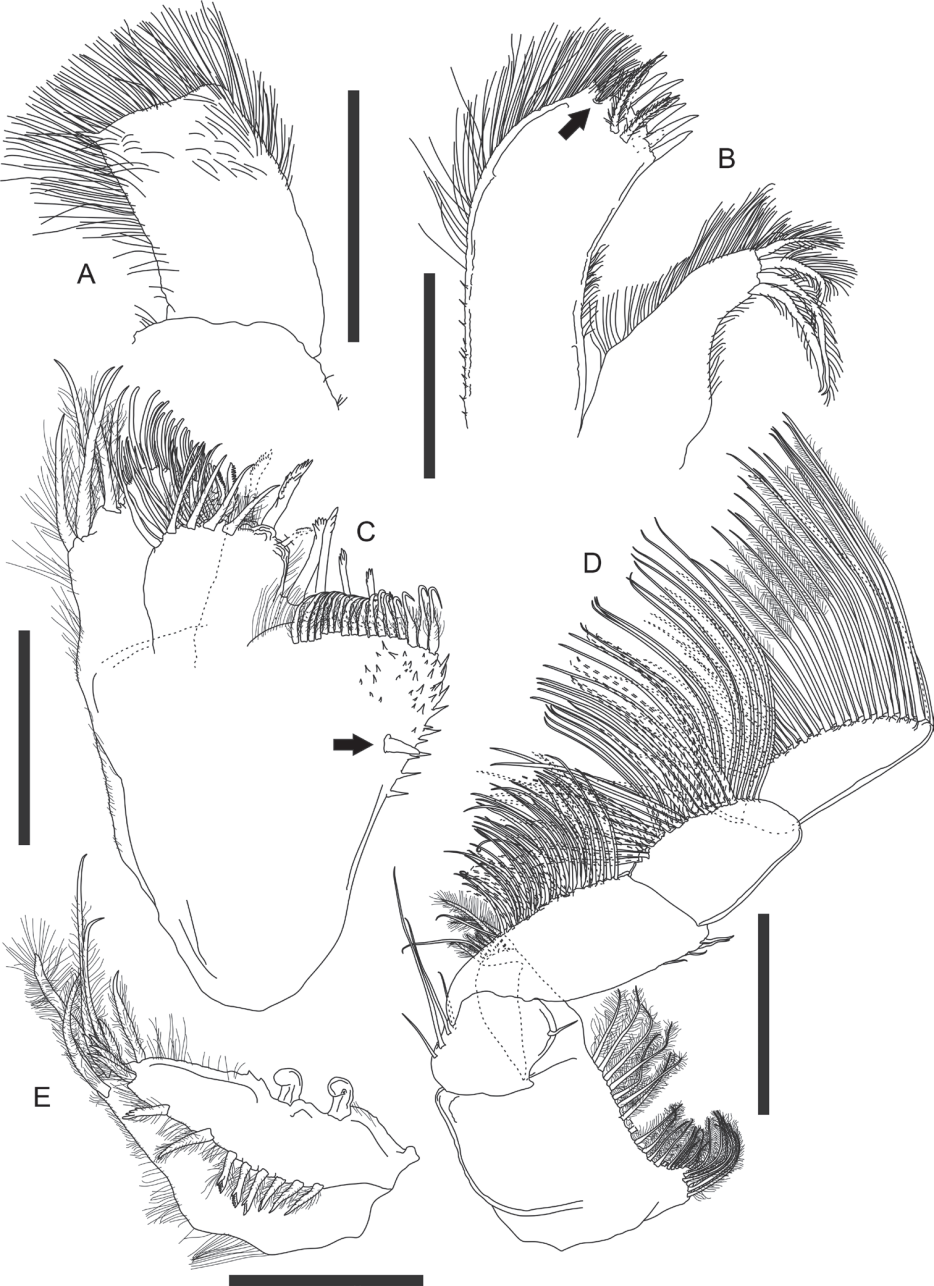
*Phoxokalliapseudesshandongensis* sp. nov. Paratype (MBM287294), non-ovigerous female **A** labium palp **B** maxillule endites **C** maxilla **D** maxilliped **E** maxilliped endite. Scale bars: 0.1 mm (**A, E**); 0.2 mm (**B–D**).

**Figure 9.**
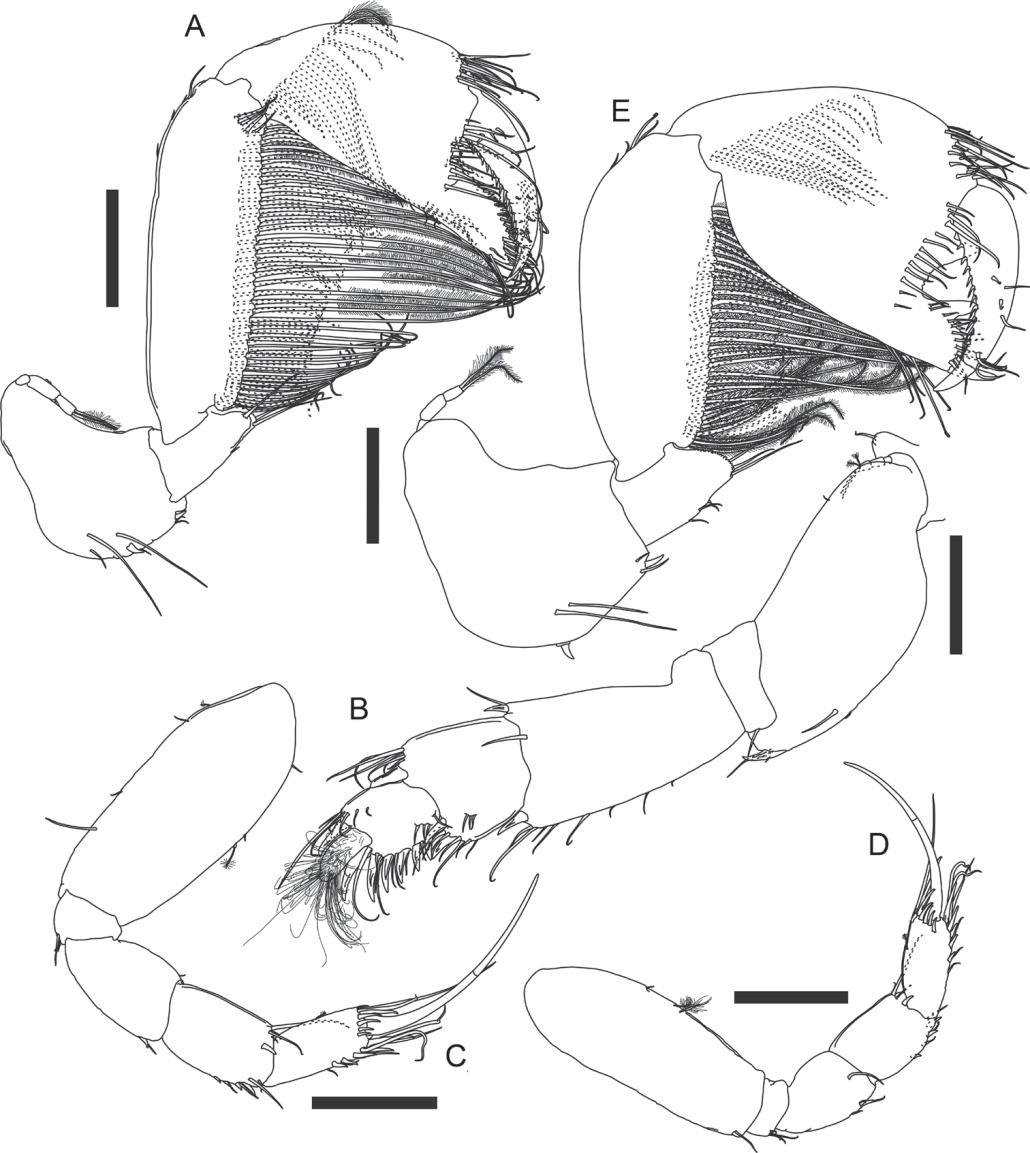
*Phoxokalliapseudesshandongensis* sp. nov. Paratype (MBM287294), female **A** right cheliped **C** right pereopod 2 **D** right pereopod 3. Paratype (MBM287296), non-ovigerous female **B** left pereopod 1. Allotype (MBM287295), male **E** right cheliped. Scale bars: 0.2 mm (**A–E**).

**Figure 10.**
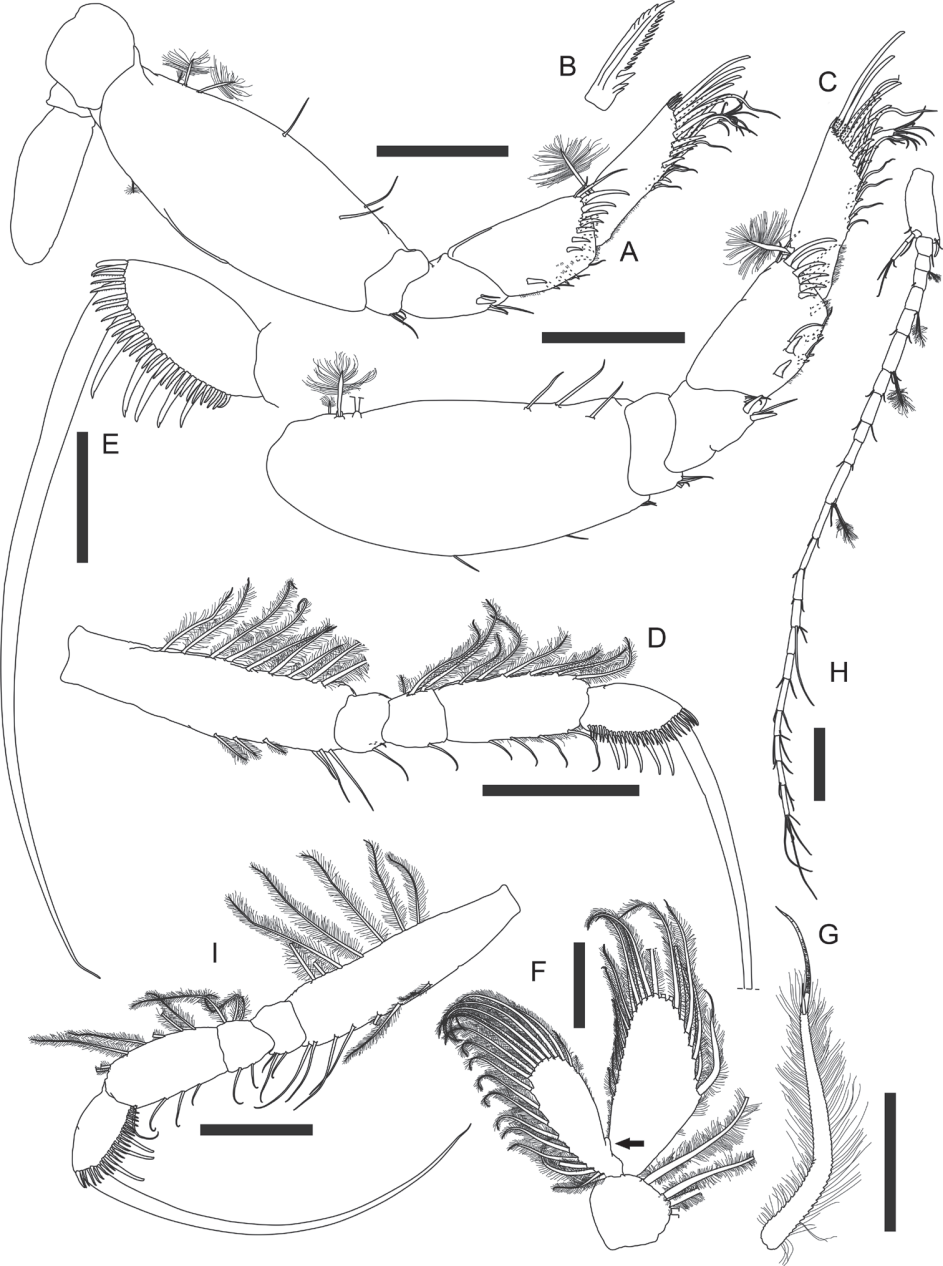
*Phoxokalliapseudesshandongensis* sp. nov. Paratype (MBM287294), non-ovigerous female **A** right pereopod 4 **B** dorsodistal spiniform seta on pereopod 4 propodus **C** right pereopod 5 **D** right pereopod 6 **E** left pereopod 6 propodus and dactylus **F** left pleopod 2 **G** pleopod endopod innermost seta. Paratype (MBM287296), non-ovigerous female **H** right uropod. Allotype (MBM287295), male **I** left pereopod 6. Scale bars: 0.2 mm (**A, C, D, H, I**); 0.1 mm (**E, F, G**).

Figs 6 , 7 , 8 , 9 , 10 , Table 2

##### Type material.

***Holotype***: MBM287298, one non-ovigerous female, 6.8 mm; the Yellow Sea, 19 June 2019, from sand-gravel substrate at depth of 33 m, 35°59'N, 121°00'E. ***Allotype***: MBM287295, male, 6.1 mm, partially dissected and body parts preserved in 75% alcohol; Jiaozhou Bay, Qingdao, Shandong Province, China, 4 February 2018, from muddy substrate with shell fragments at depth of 4 m, 36°06'N, 120°17'E. ***Paratypes***: MBM287294, one non-ovigerous female, 6.9 mm, completely dissected and body parts preserved in 75% alcohol; Jiaozhou Bay, Qingdao, Shandong Province, China, 20 February 2004, from mud-sandy substrate at depth of 5 m, 36°02'N, 120°14'E. MBM287296, one non-ovigerous female, 6.5 mm, partially dissected and body parts preserved in 75% alcohol; Jiaozhou Bay, Qingdao, Shandong Province, China, 5 February 2007, from muddy substrate at depth of 3 m, 36°10'N, 120°20'E. MBM287297, one non-ovigerous female and one male; Jiaozhou Bay, Qingdao, Shandong Province, China, 9 August 2004, from muddy substrate at depth of 6 m, 36°02'N, 120°14'E. MBM287299, one non-ovigerous female, 6.5 mm; the Yellow Sea, 26 November 2019, sand-gravel substrate with shell fragments at depth of 36 m, 34°59'N, 120°59'E. MBM287300, four males; the Yellow Sea, 26 November 2019, muddy substrate at depth of 38 m, 34°59'N, 121°30'E.

##### Other material.

MBM147028, one female; Jiaozhou Bay, Qingdao, Shandong Province, China, 2 August 1964, from muddy substrate at depth of 7 m, 36°08'N, 120°15'E. MBM032073, four males; the Yellow Sea, 23 January 1959, from mud-sandy substrate at depth of 41 m, 38°45'N, 122°43'E. MBM147063, one male and one female; Jiaozhou Bay, Qingdao, Shandong Province, China, 4 August 1964, from mud-sandy substrate at depth of 21 m, 36°08'N, 120°15'E. MBM146415, two females; Jiaozhou Bay, Qingdao, Shandong Province, China, 4 August 1964, from muddy substrate at depth of 7 m, 36°08'N, 120°15'E. MBM146430, one female; Jiaozhou Bay, Qingdao, Shandong Province, China, 2 August 1964, from muddy substrate at depth of 9 m, 36°08'N, 120°15'E. MBM146420, one female; Jiaozhou Bay, Qingdao, Shandong Province, China, 4 August 1964, from mud-sandy substrate with gravels at depth of 23 m, 36°08'N, 120°15'E. MBM146419, one female; same collection data as MBM147028. MBM147100, two females; Jiaozhou Bay, Qingdao, Shandong Province, China, 18 August 1964, from muddy substrate at depth of 5 m, 36°08'N, 120°15'E. MBM030026, one female, Yellow Sea, 20 October 1959, from muddy substrate at depth of 17 m, 37°51'N, 121°58'E.

##### Type locality.

Jiaozhou Bay, Qingdao, Shandong Province, China, and adjacent Yellow Sea area.

##### Etymology.

The name is derived from Shandong, where the species was collected around Shandong Peninsula.

##### Diagnosis.

***Non-ovigerous female. Antennule*** peduncle article 1 inner margin with row of five spiniform setae on distal half, and two long ventral subdistal spiniform setae. ***Antenna*** peduncle article 1 inner extension with six distal plumose setae. ***Maxilla*** with one small spiniform seta near outer margin. ***Cheliped*** basis with two small ventral spiniform setae. ***Pereopod 1*** propodus with two dorsal and six ventral spiniform setae. ***Pereopod 6*** propodus with five long ventral spiniform setae; dactylus long, 3.8× as long as propodus. *Pleopod* exopod inner margin with one subproximal apophysis. ***Uropod*** basal article with one inner distal spiniform seta. ***Male*.** Similar to female except ***pereonites 2–5*** with one hyposphenia, and one robust spiniform seta on each hyposphenia. ***Each pleonite*** with one hyposphenia. ***Antennule*** peduncle article 1 robust, with one ventral spiniform seta; inner flagellum 5-articled. ***Cheliped*** fixed finger incisive margin with two large triangular apophyses. ***Pereopod 6*** propodus with three or four long ventral spiniform setae; dactylus very long, 4.6× as long as propodus.

##### Description.

**Female (non-ovigerous paratype MBM287294). *Body*** (Fig. 6A) dorsoventrally flattened, 6.9 mm long, 5.8× as long as broad. ***Carapace*** less than 0.2× as long as total body length, as long as broad, rostrum rounded, lateral margin with one simple seta midway. ***Pereon*** half as long as total body length, each pereonite in similar width, slightly broader than carapace, anterolateral margin with one simple seta and several setules; pereonites 1–6 0.5, 0.5, 0.6, 0.8, 0.7, 0.4× as long as broad, respectively. ***Pleon*** 0.2× as long as total body length, each pleonite in similar width, slightly broader than pereonites, with eight or nine plumose setae on blunt lateral margin, one anterolateral simple seta on dorsal surface. ***Pleotelson*** posteriorly tapering and distally bifurcate, *ca.* as long as three pleonites combined, slightly broader than long, with two distal plumose setae.

***Antennule*** (Fig. 7A) peduncle article 1 ~ 0.4× as long as total antennule length, 2.7× as long as broad, with ten simple setae and four broom setae along outer margin, inner margin with six simple setae on proximal half, two simple setae, and a row of five spiniform setae on distal half, and two long ventral subdistal spiniform setae; article 2 0.3× as long as peduncle article 1, 1.7× as long as broad, distal half with three outer, five inner simple setae, and two ventral broom setae; article 3 half as long as article 2, with three distal simple setae; article 4 0.7× as long as article 3, with one distal simple seta; outer flagellum longer than peduncle article 1, 11-articled, each article with at most four distal simple setae, articles 4, 6, and 7 each with one distal aesthetasc; inner flagellum 6-articled, articles 1–4 each with one distal simple seta, article 5 naked, article 6 with three distal setae.

***Antenna*** (Fig. 7B) article 1 outer margin with one distal simple seta, inner extension long, covered with setules, with six distal plumose setae; article 2 inner margin covered with setules, squama with six simple setae along outer to distal margin, and spinules along inner margin; article 3 very short, with two inner plumose setae; article 4 nearly half as long as antenna, with one outer broom seta midway, a row of ten plumose setae along inner margin, and a row of 20 ventral plumose setae; article 5 inner and outer margin both with four simple setae, distal margin with five dorsal simple setae and two ventral plumose setae; articles 6 and 7 both with five distal simple setae and two ventral plumose setae; article 8 with one distal simple and two ventral plumose setae; article 9 with two ventral plumose setae; article 10 with five distal simple setae.

***Labrum*** (Fig. 7C, figure and description based on non-ovigerous female paratype, MBM287296, 6.5 mm long) distal margin rounded, covered with numerous setules; clypeus with two robust and two smaller cusps.

***Left mandible*** outer margin with one conspicuous apophysis; both incisor and lacinia mobilis (Fig. 7D, figure based on MBM287296) distal margin denticulate; setal row with five serrate setae; molar distal margin crenulate; palp with 29 long plumose setae along inner margin and one distal spiniform seta. Right mandible (Fig. 7E) similar to left mandible but without lacinia mobilis, incisor (Fig. 7F, figure based on MBM287296) with two large distal denticles.

***Labium*** palp (Fig. 8A) subrectangular, 1.5× as long as broad, covered with setules, with one small spine-like apophysis on inner distal corner.

***Maxillule*** (Fig. 8B) inner endite covered with numerous long setules, with four distal plumose setae; outer endite covered with numerous long setules, with a row of spinules along outer margin, ten spiniform setae on distal margin, two subdistal plumose setae, and one distinct concavity (arrow) on outer distal corner.

***Maxilla*** (Fig. 8C) outer lobe of movable endite with five plumose setae; inner lobe of movable endite with row of 15 distally blunt setae, and two comb-like setae along distal margin; outer lobe of fixed endite with a row of 15 simple setae, four plumose setae, and two robust and one smaller serrate seta; inner lobe of fixed endite with five distally serrate setae and a row of 17 plumose setae; outer margin and sub-outer surface covered with spinules, with one sub-outer spiniform seta (arrowed).

***Maxilliped*** (Fig. 8D) basis with a row of 25 plumose setae along outer margin; endite (Fig. 8E) covered with setules entirely, with ten plumose setae on distal margin, two robust coupling hooks on inner margin, and eight setulose and distally serrate setae on inner fold; palp article 1 with four simple setae on inner margin and one simple seta on outer margin; article 2 with two rows of numerous long plumose setae along inner margin, and four subdistal simple setae on outer margin; articles 3 and 4 both with two rows of numerous long plumose setae along inner margin.

***Epignath*** not examined.

***Cheliped*** (Fig. 9A) exopod 3-articled, article 3 with two distal plumose setae; basis ~ 1.5× as long as broad, ventral margin with one spiniform seta midway, three meddle simple setae, one subdistal spiniform seta, and two subdistal simple setae; merus with one ventral simple seta, anterior corner protruded and rounded, anterior margin crenulate, with three simple setae; carpus ~ 3.5× as long as broad, with two rows of long plumose setae along ventral margin, both rows with ~ 40 setae, dorsal margin with three short and one longer simple setae on distal half, anteroventral corner with four outer simple setae; propodus and fixed finger combined nearly as long as carpus, 2.2× as long as broad, palm 1.6× as long as broad, with a row of 15 inner plumose setae and a cluster of 11 simple setae on anterodorsal corner near insertion of dactylus, fixed finger with 17 simple setae on ventral margin or subventral surface, 17 simple setae and an intensive row of small denticles along incisive margin, a row of 12 outer and four inner simple setae near insertion of dactylus; dactylus plus unguis 4.5× as long as broad, slightly curved, with nine outer and four inner simple setae, and a row of 15 small spiniform setae along incisive margin.

***Pereopod 1*** (Fig. 9B, figure and description based on MBM287296) exopod 3-articled, article 3 with two distal plumose setae; coxa with one simple seta on blunt apophysis; basis ~ 2.2× as long as broad, dorsal margin with one simple and two broom setae on proximal half, ventral margin with one midway, two subdistal simple setae, and one robust subdistal spiniform setae, and one simple seta on subventral surface; ischium with one ventral simple seta; merus 0.8× as long as basis, 1.9× as long as broad, distal margin with one dorsal simple seta, one dorsal spiniform seta, one outer simple seta, and one ventral spiniform seta, ventral margin with eight simple setae; carpus short, *ca.* half as long as merus, slightly longer than broad, dorsal margin with a cluster of six subdistal simple setae and one robust distal spiniform seta, ventral margin with seven subdistal simple setae and two spiniform setae, outer surface with a cluster of three short simple setae; propodus 0.7× as long as carpus, 1.3× as long as broad, dorsal margin with a row of seven subdistal simple setae and two distal spiniform setae in unequal size, ventral margin with eight simple setae and six spiniform setae, outer surface with four short subproximal simple setae; dactylus 0.7× as long as propodus, covered with intensive long sensory setae.

***Pereopod 2*** (Fig. 9C) basis 2.5× as long as broad, dorsal margin with two simple setae and one broom seta, ventral margin three simple setae and one broom seta; ischium with three ventrodistal simple setae; merus 0.4× as long as basis, 1.7× as long as broad, dorsal margin with one subdistal simple seta, ventral margin with one subdistal simple seta and one subdistal spiniform seta; carpus slightly shorter than merus, 1.5× as long as broad, dorsal margin with a cluster of two longer and two short distal simple setae, and one outer distal spiniform seta, ventral margin with four simple setae and four spiniform setae on distal half; propodus slightly shorter than carpus, 2.1× as long as broad, with one dorsal broom seta midway, and a row of two distal simple setae and six spiniform setae, ventral margin with three long and three shorter simple setae, and a row of five spiniform setae on distal half; dactylus plus unguis long and slender, 2.2× as long as propodus, without sensory lobe.

***Pereopod 3*** (Fig. 9D) basis 2.7× as long as broad, dorsal margin with one simple and two broom setae midway, ventral margin with one distal simple seta and one distal spiniform seta; ischium with four ventrodistal simple setae; merus 0.4× as long as basis, 1.6× as long as broad, ventral margin with three simple setae and two spiniform setae on distal half, outer surface with one subdistal simple seta; carpus slightly shorter than merus, 1.4× as long as broad, dorsal margin with one distal spiniform seta and three distal simple setae, ventral margin with three simple setae and four spiniform setae on distal half; propodus and dactylus similar to those of pereopod 2.

***Pereopod 4*** (Fig. 10A) basis 2.4× as long as broad, dorsal margin with three subproximal broom setae, one midway and two subdistal simple setae, ventral margin with one subproximal broom seta; ischium with four ventrodistal simple setae; merus short, 0.3× as long as basis, 1.3× as long as broad, ventral margin with three subdistal simple setae and two subdistal spiniform setae; carpus 1.4× as long as merus, 1.9× as long as broad, with two dorsodistal simple setae, a row of eight outer spiniform setae, a row of eight inner spiniform setae, and three ventral simple setae, ventral half covered with setules; propodus slightly shorter than carpus, 2.7× as long as broad, with one large dorsoproximal broom seta and a row of five small dorsodistal leaf-like spiniform setae (Fig. 10B), a row of eight outer, eight inner spiniform setae and six simple setae along ventral to distal margin, ventral margin covered with setules; dactylus 0.4× as long as propodus, with nine distal simple setae.

***Pereopod 5*** (Fig. 10C) basis 2.5× as long as broad, dorsal margin with three broom setae on proximal half and three simple setae on distal half, ventral margin with a cluster of three distal simple setae, and two simple setae on distal half; ischium, merus, propodus and dactylus very similar to those of pereopod 4 but merus with seven inner spiniform setae.

***Pereopod 6*** (Fig. 10D) basis 3.7× as long as broad, with nine dorsal and three ventral plumose setae, and three ventral subdistal simple setae; ischium with two ventrodistal simple setae; merus short, 0.2× as long as basis, slightly as long as broad, with three dorsal plumose setae and one ventral simple seta; carpus 2.7× as long as merus, 2.4× as long as broad, with six dorsal plumose setae and four ventral simple setae, ventral margin covered with setules; propodus (Fig. 10E) ~ 0.7× as long as carpus, almost twice as long as broad, with five long ventral spiniform setae, a row of 27 short serrate setae along ventral to distal margin, and one dorsodistal spiniform seta; dactylus (Fig. 10E) long and slender, 3.8× as long as propodus.

***Pleopods*** (Fig. 10F) basal article with four inner plumose setae; exopod with 20 plumose setae, inner margin with one subproximal protrusion (arrowed); endopod with 22 plumose setae, innermost seta (Fig. 10G) robust, distally branched and ringed.

***Uropod*** (Fig. 10H, figure and description based on MBM287296) basal article 2.7× as long as broad, with two outer, one inner distal simple seta, and one inner distal spine; exopod 3-articled, article 3 with four distal simple setae; endopod 22-articled, each article naked or with at most five distal simple setae, article 2 with three distal broom setae, articles 4, 6, and 11 each with two distal broom setae.

**Male (allotype MBM287295).** Similar to female with following variations: ***body*** (Fig. 6B) less slender, 6.1 mm long, 4.8× as long as broad. ***Carapace*** ~ 0.2× as long as total body length, slightly broader than long, lateral margin with one simple seta midway. ***Each pereonite*** with 1–3 anterolateral simple setae, pereonites 1–5 each with one hyposphenia (Fig. 6C), and one robust spiniform seta on each hyposphenia, pereonite 6 with genital cone; pereonite 1 conspicuously broader than subsequent pereonites, 1.2× as broad as pereonite 2, 0.4× as long as broad; pereonites 2–6 in similar width, relatively short, 0.5, 0.5, 0.5, 0.5, 0.3× as long as broad respectively. ***Each pleonite ca.*** as wide as pereonite 1, with one hyposphenia, one dorsolateral simple seta and 6–10 lateral plumose setae.

***Antennule*** (Fig. 7G) peduncle article 1 robust, 0.4× as long as total antennule length, 2.2× as long as broad, outer margin with eight simple and five broom setae, inner margin seven simple and three broom setae, and one small ventral spiniform seta near inner distal corner; article 2 0.4× as long as article 1, 1.4× as long as broad, with six inner simple setae, two simple and two broom ventral setae; peduncle article 3 0.5× as long as peduncle article 2, slightly as long as broad, with one outer subdistal and three inner distal simple setae; peduncle article 4 0.7× as long as article 3, distal margin with two inner simple setae; outer flagellum longer than peduncle article 1, 11-articled, article 1 with 15 distal aesthetascs; article 2 with ~ 15 distal aesthetascs and two distal simple setae, article 3 with 11 distal aesthetascs and two distal simple setae, articles 4 and 5 both with six distal aesthetascs and two distal simple setae, articles 6, 7 and 9 each with one distal aesthetasc and two distal simple setae, articles 8 and 10 naked, article 11 with four distal simple setae; inner flagellum 5-articled, articles 1–3 each with one distal simple seta, article 4 with two distal simple setae, article 5 with four distal simple setae.

***Antenna*** article 1 inner extension with six distal plumose setae.

***Cheliped*** (Fig. 9E) exopod 3-articled, article 3 with two distal plumose setae; basis ~ 1.4× as long as broad, with two long outer setae, ventral margin with one spiniform seta midway, two distal short simple setae and one distal spiniform seta; merus with three ventral simple setae, anterior corner protruced and rounded, anterior margin crenulate, with three simple setae; carpus 2.6× as long as broad, dorsal margin with four subdistal simple setae, ventral margin with two rows of long plumose setae, outer row with 35 plumose setae, inner row with 32 plumose setae; propodus large and robust, combined with fixed finger nearly as long as carpus, 1.8× as long as broad, palm 1.3× as long as broad, with a row of 15 inner plumose setae, a cluster of 17 anterodorsal simple setae and a row of 13 outer simple setae near insertion of dactylus, fixed finger with five ventral simple setae, incisive margin with two strong triangular apophyses, a row of eleven simple setae, and a row of seven small spiniform setae; dactylus plus unguis 3.7× as long as broad, slightly curved, dorsoproximally expanded, with nine outer setae and a row of ten small spiniform setae along incisive margin.

***Pereopod 6*** (Fig. 10I) basis ~ 4.1× as long as broad, dorsal margin with seven plumose setae, ventral margin with three plumose setae midway and four subdistal simple setae; ischium with three ventral subdistal simple setae; merus short, 0.2× as long as basis, slightly longer than broad, with three dorsal plumose and two ventral simple setae; carpus 2.3× as long as merus, 2.1× as long as broad, with seven dorsal plumose and four ventral simple setae; carpus 0.7× as long as merus, 1.8× as long as broad, with three (on left pereopod 6) or four (on right pereopod 6) long ventral spiniform setae, a row of 24 short serrate setae along ventral to distal margin, and one dorsodistal spiniform seta; dactylus very long and slender, 4.6× as long as propodus.

Some other morphological characters of males given in Table 2.

**Table 2.** Morphological comparison among all species of *Phoxokalliapseudes*, modified and updated from Wi et al. (2017) and differentiating between female (F) and male (M); T = triangular, S = square, R = rounded, N = no, Y = yes, A = absent, P = present.

Character / Species name	*P.shandongensis* sp. nov.		*P.aculeatus*		*P.cinctus*		*P.gibbus*		*P.gobinae* (Bamber)		*Phoxokalliapseudescf.gobinae* Drum and Heard		*P.multiarticulus* (Guţu)	*Phoxokalliapseudescf.multiarticulus* Drum and Heard		*P.singaporensis*	*P.tomiokaensis*	
	F	M	F	M	F	M	F	M	F	M	F	M	F	F	M	M	F	M
**Antennule**																		
Number of inner flagellum articles	6	5	6	5	4	5	5	5	-	6	4	4	8	6	6	5	6	5
**Antenna**																		
Number of setae on article 1 inner extension	6	6	7	7	5	5	6	-	-	7	-	6	-	-	8	6	7	6
Number of setae on article 3	2	2	2	2	1	2	2	-	-	0	-	2	-	-	3	1	2	2
**Cheliped**																		
Number of basis ventral spiniform setae	2	2	2	2	2	2	2	2	0	1	0	2	-	0	2	0	0	2
Propodus long/wide ratio	2.2	1.8	1.6	1.3	3.0	2.5	2.5	1.6	2.7	1.8	3.3	1.5	-	2.1	1.4	1.5	2.1	1.5
Shape of fixed finger apophyses		T		T		T		T		S		S			R	T		T
**Pereopod 1**																		
Number of propodus ventral/dorsal spiniform setae	6/2	6/2	7/2	7/2	-	7/2	9/2	-	-	7/2		6/2	4/2	5/2	-	5/2	-	6/2
**Pereopod 6**																		
Dactylus/propodus length ratio	3.8	4.6	2.6	4.4	3.1	4.3	3.5	4.5	-	4.1	-	4.7	-	2.0	3.9	3.6	-	2.7
Number of propodus long ventral spiniform setae	5	3–4	5	5	4	4	6	-	-	2	-	3	5	5	3	2	-	5
Dactylus distally bifurcated	N	N	N	N	N	N	N	-	-	N	-	N	Y	Y	Y	Y	-	N
**Pereopods 2–3**																		
Presence of dactylus sensory organ	A	A	A	A	A	A	A	-	A	A	A	A	P	P	P	P	A	A
**References**	present study		Wi et al. 2017		Wi et al. 2017		Wi et al. 2019		Bamber 1998		Drumm and Heard 2011		Guţu 2006	Drumm and Heard 2011		Drumm and Heard 2011	Shiino 1966	

##### Remarks.

Seven species have been reported for the genus *Phoxokalliapseudes*, all from the western Pacific, i.e., *P.aculeatus* Wi, Kang & Soh, 2017 (southwestern Korea), *P.cinctus* Wi, Kang & Soh, 2017 (southern Korea), *P.gibbus* Wi, Yu & Kang, 2019 (Jeju Island, Korea), *P.gobinae* Bamber, 1998 (Brunei and Sabah; Bamber 2013), *P.multiarticulus* Guţu, 2006 (Darwin, northern Australia), *P.singaporensis* Drumm & Heard, 2011 (Singapore), and *P.tomiokaensis* Shiino, 1966 (Kyushu, Japan). The eighth species, *Phoxokalliapseudesshandongensis* sp. nov. from the present study, is geographically closest to *P.aculeatus* and *P.gibbus*.

Morphologically, *Phoxokalliapseudesshandongensis* also closely resembles *P.gibbus*, by having six distal plumose setae on inner extension of antenna article 1, two ventral spiniform setae on cheliped basis, two large triangular (conical) apophyses on incisive margin of male cheliped fixed finger, similar female and male pereopod 6 dactylus to propodus length ratio, and one subproximal protrusion on inner margin of each pleopod exopod. However, *P.shandongensis* can be distinguished from *P.gibbus* by several significant differences: 1) female antennule peduncle article 1 with two long and conspicuous ventral spiniform setae and five smaller spiniform setae on inner margin, vs. only one ventral spiniform seta in *P.gibbus*; 2) with six ventral spiniform setae on pereopod 1 propodus, vs. nine in *P.gibbus*; 3) with five ventral spiniform setae on pereopod 6 propodus, vs. six in *P.gibbus*; 4) with one inner distal spiniform seta on uropod basal article (Figs 7A, B, 9A, B, E, 10D–I, Table 2; Wi et al. 2019: figs4C, E, 5C–F, H, I, 6F, G, 7E, F, H, I).

Among *Phoxokalliapseudes* species, only *P.shandongensis* and *P.gobinae* have one inner distal spiniform seta on uropod basal article. However, the male of *P.shandongensis* have two large triangular (conical) apophyses on incisive margin of cheliped fixed finger, easily distinguished from the male of *P.gobinae* with one distal square and one triangular (conical) apophysis. In addition, the presence of two ventral spiniform setae on female cheliped basis can also differentiate the new species from *P.gobinae* (Fig. 9A, E, Table 2; Bamber 1998: fig 9A, B; Drumm and Heard 2011: figs 22F, 24D).

#### ﻿Key to the species of *Phoxokalliapseudes* (modified and updated from Wi et al. 2019; see also Table 2)

**Table:** 

1	Pereopods 2 and 3 dactylus without proximal sensory lobe; pereopod 6 dactylus without subdistal seta	**2**
–	Pereopods 2 and 3 dactylus with proximal sensory lobe; pereopod 6 dactylus with subdistal seta	**3**
2	Male cheliped fixed finger incisive margin with square-shaped apophysis	***P.gobinae***
–	Male cheliped fixed finger incisive margin with triangular (conical) apophysis	**4**
3	Antennule article 1 with three robust subdistal spiniform setae	***P.multiarticulus***
–	Antennule article 1 with one small subdistal spiniform setae	***P.singaporensis***
4	Male cheliped fixed fingered incisive margin with small triangular (conical) apophysis	***P.cinctus***
–	Male cheliped fixed fingered incisive margin with large triangular (conical) apophysis	**5**
5	Female cheliped basis without ventral spiniform seta	***P.tomiokaensis***
–	Female cheliped basis with two ventral spiniform setae	**6**
6	Female antennule article 1 with two robust and five smaller subdistal spiniform setae	***P.shandongensis*** sp. nov.
–	Female antennule article 1 with two subdistal spiniform setae	**7**
7	Antennule article 1 with two robust subdistal spiniform setae	***P.aculeatus***
–	Antennule article 1 with one robust and one small subdistal spiniform seta	***P.gibbus***

### Family Parapseudidae Guţu, 1981

**Subfamily Pakistanapseudinae Guţu, 2008**

#### ﻿Genus Swireapseudes

Taxon classificationAnimaliaTanaidaceaParapseudidae

Bamber, 1997

BF9E287B-87EC-53CD-B60E-F97B1EF45E35

##### Type species.

*Swireapseudestoloensis* Bamber, 1997

##### Remarks.

This genus has once been synonymised with *Pakistanapseudes* by Bamber and Sheader (2003) but revalidated by Guţu (2006). The main morphological features that distinguish these two genera are: 1) the presence of ventrodistal prolongation on pereopods 2, 3, 5 and 6 in species of *Swireapseudes*, compared to the presence of a ventrodistal prolongation on pereopods 2 and 3 in some species of *Pakistanapseudes* but never on pereopods 5 and 6; 2) the presence of sexual dimorphism of cheliped in species of *Swireapseudes* (Guţu 2008). Two species of *Swireapseudes* have been previously recorded at very distant locations: *Swireapseudesbirdi* Guţu & Iliffe, 2008 (Eleuthera Island, Bahamas) and *Swireapseudestoloensis* Bamber, 1997 (Tolo Channel, Hong Kong).

#### ﻿ Swireapseudes planafrontis sp. nov.

Taxon classificationAnimaliaTanaidaceaApseudidae

6B7F4611-94CF-51F1-946D-D81D7591AEEA

http://zoobank.org/D8634839-52D1-4117-B015-442224608B68

**Figure 11.**
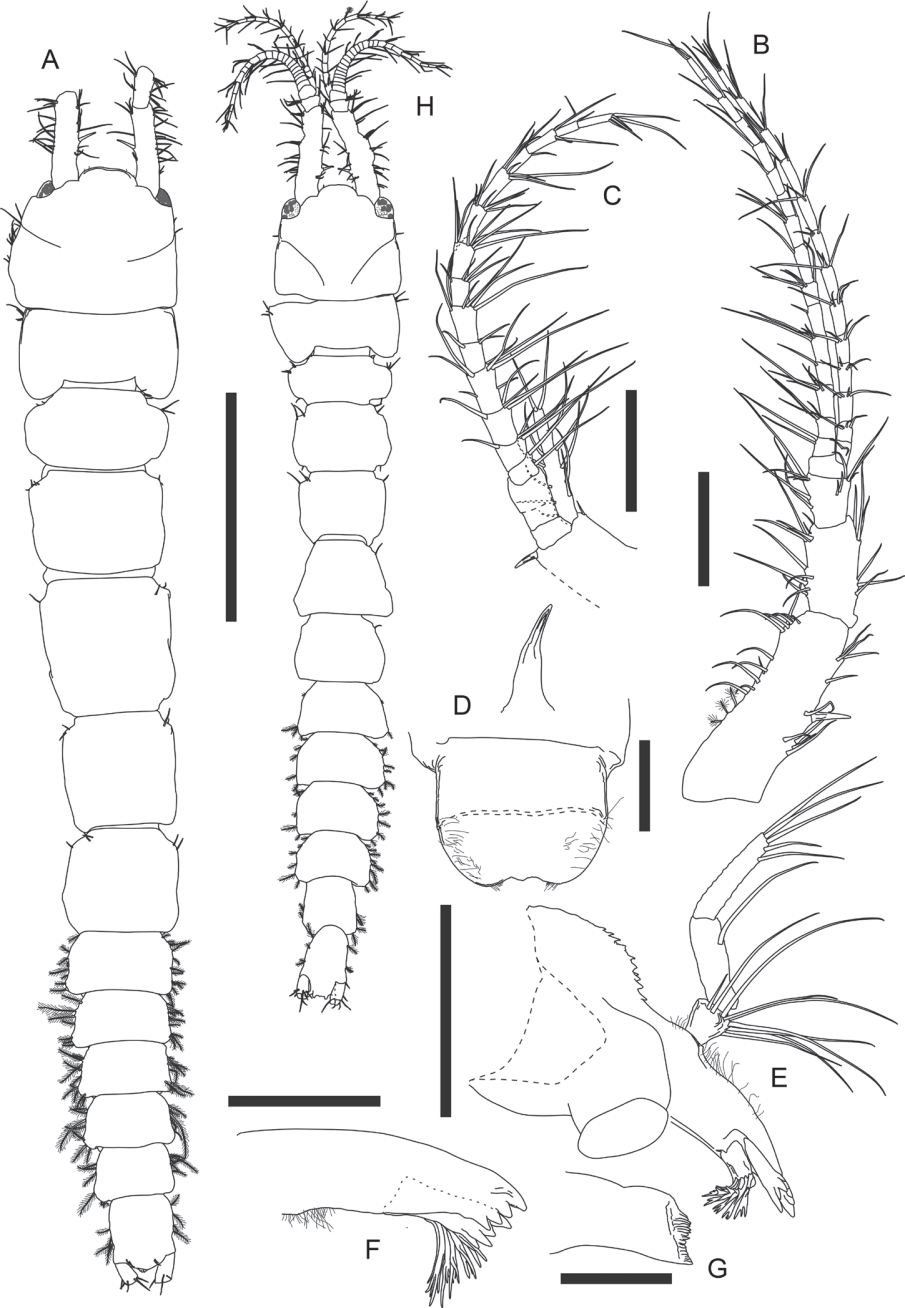
*Swireapseudesplanafrontis* sp. nov. Paratype (MBM287303), non-ovigerous female **A** body dorsal view. Paratype (MBM287304), non-ovigerous female **B** left antennule. Paratype (MBM287301), ovigerous female **C** right antenna **D** epistome and labrum **E** left mandible **F** left mandible incisor, lacinia mobilis and setal row **G** left mandible molar. Allotype (MBM287302), male **H** body dorsal view. Scale bars: 2 mm (**A, H**); 0.2 mm (**B, C, E**); 0.1 mm (**D, F, G**).

**Figure 12.**
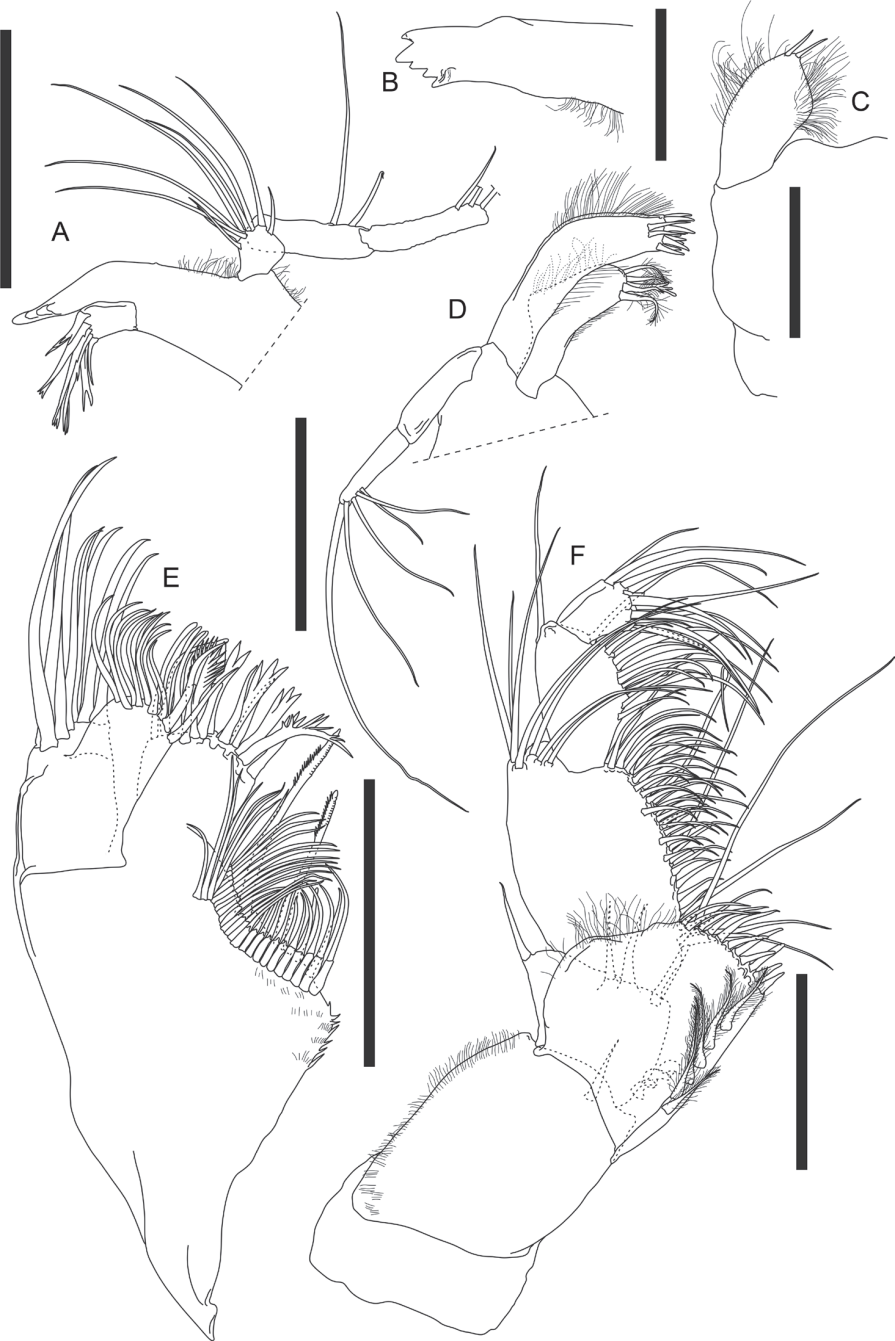
*Swireapseudesplanafrontis* sp. nov. Paratype (MBM287301), ovigerous female **A** right mandible **B** right mandible incisor **C** labium **D** maxillule **E** maxilla **F** maxilliped. Scale bars: 0.2 mm (**A, D**); 0.1 mm (**B, C, E, F**).

**Figure 13.**
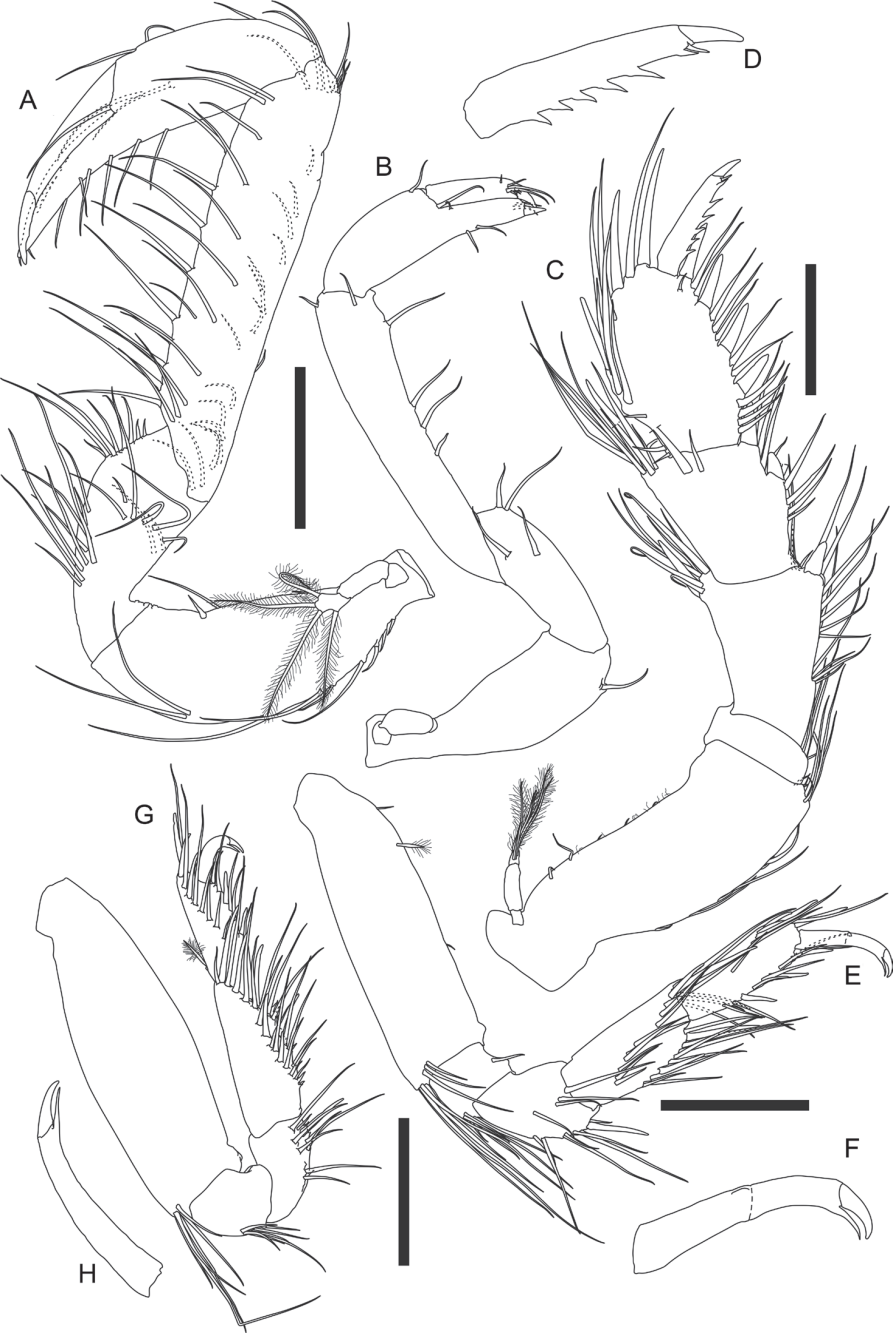
*Swireapseudesplanafrontis* sp. nov. Paratype (MBM287301), ovigerous female **A** left cheliped **B** right cheliped **C** right pereopod 1 **D** right pereopod 1 dactylus **E** right pereopod 2 **F** right pereopod 2 dactylus **G** right pereopod 3 **H** right pereopod 3 dactylus. Scale bars: 0.2 mm (**A–C, E, G**).

**Figure 14.**
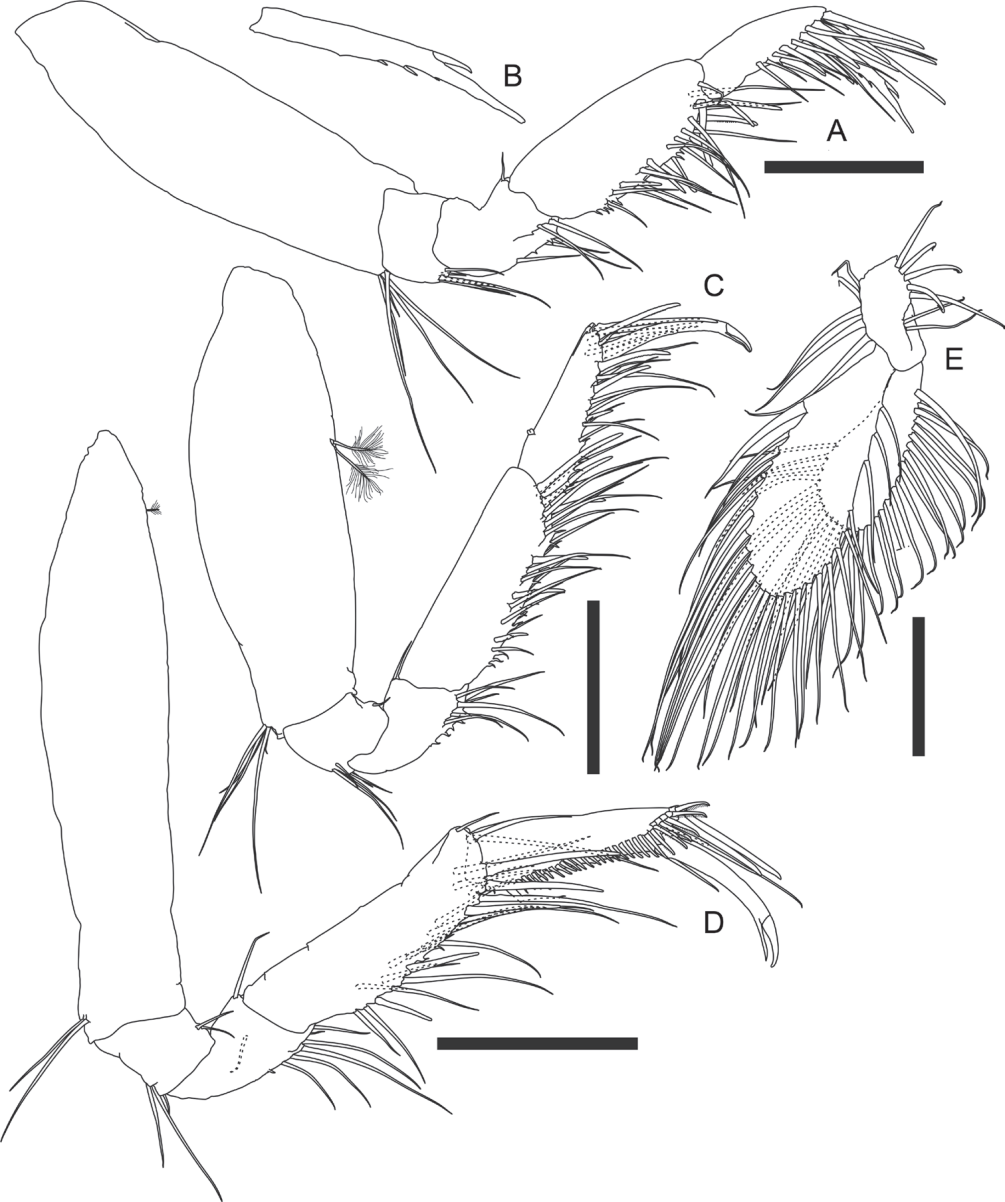
*Swireapseudesplanafrontis* sp. nov. Paratype (MBM287305), non-ovigerous female **A** right pereopod 4 **B** right pereopod 4 dactylus. Paratype (MBM287301), ovigerous female **C** right pereopod 5 **D** right pereopod 6 **E** right pleopod 1 (all setules omitted). Scale bars: 0.2 mm (**A, C–E**).

**Figure 15.**
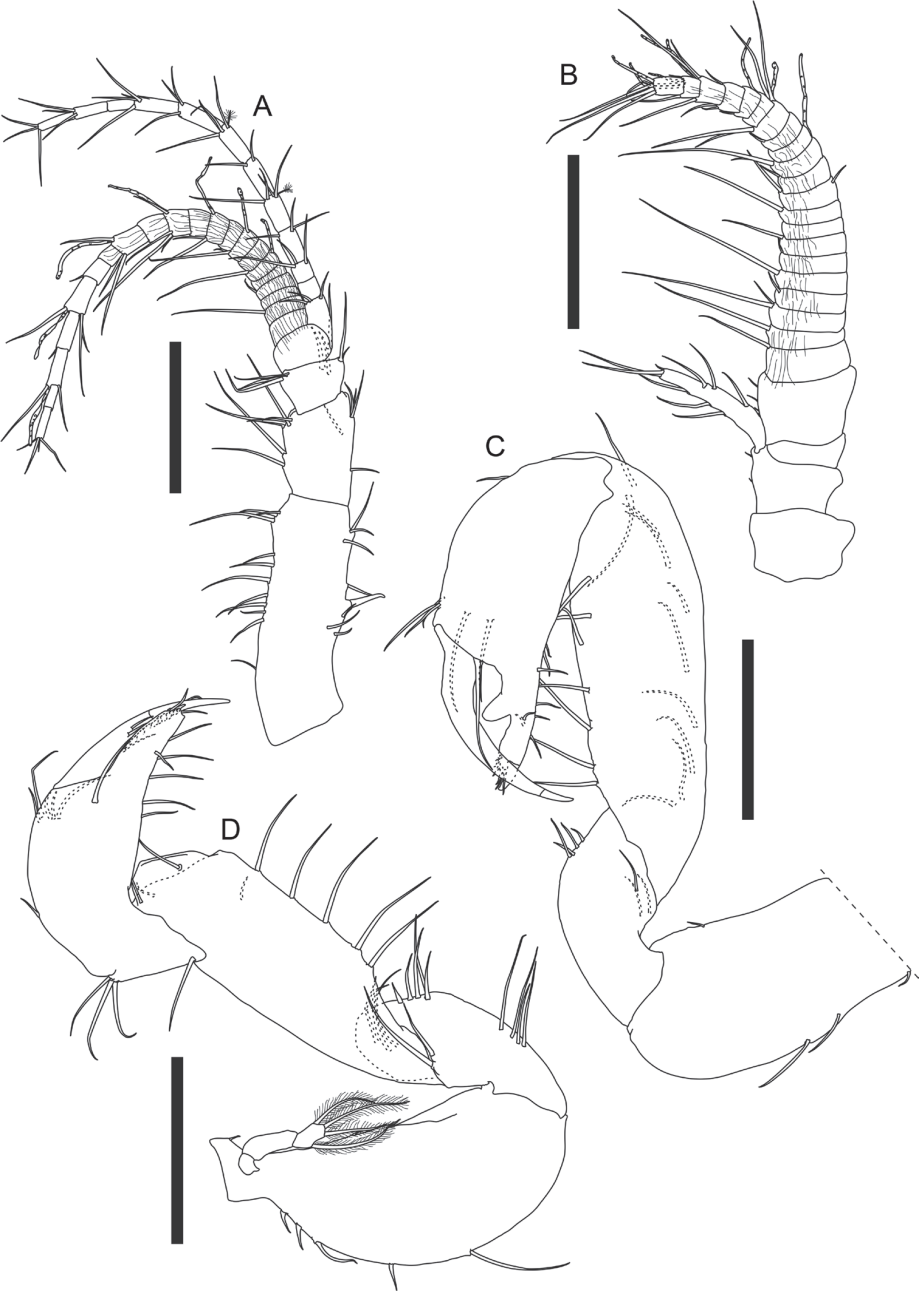
*Swireapseudesplanafrontis* sp. nov. Allotype (MBM287302), male **A** left antennule **B** left antenna **D** right cheliped. Paratype (MBM287307), male **C** left cheliped. Scale bars: 0.2 mm.

Figs 11 , 12 , 13 , 14 , 15 , Table 3

##### Type material.

***Holotype***: MBM287312, non-ovigerous female, 6.8 mm; Jiaozhou Bay, Qingdao, Shandong Province, China, 20 November 2003, from mud-sandy substrate with shell fragments at depth of 10 m, 36°09'N, 120°19'E. ***Allotype***: MBM287302, male, 5.0 mm, completely dissected and body parts preserved in 75% alcohol; Jiaozhou Bay, Qingdao, Shandong Province, China, 15 March 2021, from muddy substrate with shell fragments at depth of 6 m, 36°06'N, 120°17'E. ***Paratypes***: MBM287301, one ovigerous female, 6.9 mm, completely dissected and body parts preserved in 75% alcohol; Jiaozhou Bay, Qingdao, Shandong Province, China, 12 November 2020, from muddy substrate at depth of 6 m, 36°05'N, 120°10'E. MBM287303, one non-ovigerous female, 6.8 mm, partially dissected and body parts preserved in 75% alcohol; same collection data as allotype. MBM287304, one non-ovigerous female, 6.8 mm, partially dissected, and body parts preserved in 75% alcohol; same collection data as allotype. MBM287305, one non-ovigerous female, 7.1 mm, partially dissected and body parts preserved in 75% alcohol; Jiaozhou Bay, Qingdao, Shandong Province, China, 11 November 2020, from muddy substrate with shell fragments at depth of 4 m, 36°06'N, 120°17'E. MBM287306, one male, 5.2 mm, partially dissected and body parts preserved in 75% alcohol; same collection data as allotype. MBM287307, one male, 4.2 mm, partially dissected and body parts preserved in 75% alcohol; same collection data as allotype. MBM287308, one male, 4.2 mm; same collection data as allotype. MBM287309, one non-ovigerous female, 5.0 mm; Jiaozhou Bay, Qingdao, Shandong Province, China, 3 February 2021, from muddy substrate at depth of 14 m, 36°06'N, 120°15'E. MBM287310, one ovigerous female, 6.0 mm; same collection data as paratype MBM287305. MBM287311, one non-ovigerous female, 7.8 mm; same collection data as allotype.

##### Other material.

MBM147029, two females, Jiaozhou Bay, Qingdao, Shandong Province, China, 2 August 1964, from muddy substrate at depth of 7 m, 36°08'N, 120°15'E.

##### Type locality.

Jiaozhou Bay, Qingdao, Shandong Province, China.

##### Etymology.

The name is derived from the Latin *planula* (flat) and *frontis* (forehead), referring to the wide and flat rostrum.

##### Diagnosis.

***Ovigerous female*. *Rostrum*** rounded, with wide and flat distal margin. ***Antennule*** article 1 inner margin with one strong spiniform seta midway. ***Antenna*** article 2 with one inner distal spiniform seta. ***Labium*** palp without inner distal expansion. ***Cheliped*** slightly dimorphic; left cheliped more setose; right cheliped more slender, exopod article 3 small and naked. ***Pereopod 1*** merus with one short and strong ventrodistal spiniform seta; carpus with two short and strong ventral spiniform setae; dactylus with six pointed ventral denticles and one small ventrodistal spiniform seta, together with unguis subchela-like. ***Pereopods 2, 3, 5, and 6*** with chela-like dactylus. ***Pereopod 4*** dactylus with particularly long ventrodistal prolongation, > 2× as long as unguis. **Male.** Similar to female except: ***Antennule*** outer flagellum proximal articles short and wide, covered with numerous aesthetascs. ***Antenna*** article 2 without inner distal spiniform seta, flagellum inner half covered with numerous aesthetascs, proximal articles very wide and short, distal articles narrower. ***Cheliped*** relatively robust; left and right cheliped dimorphic or not; fixed finger incisive margin with or without one large and blunt apophysis.

##### Description.

**Female (ovigerous paratype MBM287301). *Body*** (Fig. 11A, figure and description based on non-ovigerous female paratype MBM287303) elongate, dorsoventrally flattened, 6.8 mm long, 6.6× as long as broad, posteriorly narrower. ***Carapace*** subrectangular, ~ 0.1× as long as total body length, 0.9× as long as broad, rostrum wide and rounded, anterior margin flat, with four short simple setae, lateral margin with several simple setae. ***Eye lobe*** well separated, pigmented. ***Pereon*** ~ 0.6× as long as total body length; pereonite 1 broadest, 0.6× as long as broad; pereonite 2 shortest, 0.9× as long as pereonite 1, 0.6× as long as broad; pereonite 3 1.3× as long as pereonite 2, 0.8× as long as broad; pereonite 4 longest, 1.4× as long as pereonite 3, *ca.* as long as broad; pereonite 5 0.8× as long as pereonite 4, *ca.* as long as broad; pereonite 6 0.9× as long as pereonite 5, 0.9× as long as broad, each pereonite with a few anterolateral simple setae. ***Pleon*** ~ 0.25× body length, posteriorly narrower; each pleonite rectangular and similar in length, with several lateral plumose setae. ***Pleotelson*** subrectangular, 1.4× as long as one pleonite, narrower than pleonite 5, 1.2× as long as broad, with four lateral plumose setae, posterior margin rounded, with two pairs of simple setae.

***Antennule*** (Fig. 11B, figure and description based on non-ovigerous female paratype MBM287304, 6.8 mm long) peduncle article 1 2.8× as long as broad, outer margin with three broom and 12 simple setae, inner margin with nine simple setae and one strong spiniform seta midway; article 2 half as long as article 1, twice as long as broad, with 11 outer setae and six inner simple setae; article 3 short, half as long as article 2, 1.2× as long as broad, with seven outer and six inner simple setae; article 4 naked; outer flagellum 16-articled, longer than peduncle, each article naked or with at most five distal simple setae; inner flagellum 13-articled, slightly shorter than outer flagellum, each article with two to five distal simple setae.

***Antenna*** (Fig. 11C) peduncle article 1 not examined; article 2 proximal half not examined, distal margin with one short outer simple seta and one inner spiniform seta, squama elongate, with five outer, five inner, and two distal simple setae; article 3 short and naked; articles 4 and 5 not well separated, naked; flagellum 14-articled, each article with 1–8 setae.

***Labrum*** (Fig. 11D) rounded, distal margin slightly concave, covered with setules; epistomal apophysis present.

***Left mandible*** (Fig. 11E) outer margin denticulate and covered with setules; incisor and lacinia mobilis (Fig. 11F) four-denticled, setal row with one simple and five trifurcate or multifurcate setae; molar (Fig. 11G) crenulate; palp 3-articled, article 1 short, 0.9× as long as broad, with nine simple setae along distal and inner margin, article 2 2.5× as long as article 1, with one simple seta on inner margin, article 3 *ca.* as long as article 2, with five distal simple setae. Right mandible (Fig. 12A) outer margin covered with setules; incisor (Fig. 12B) five-denticled, without lacinia mobilis, setal row similar to that of left mandible; molar not examined; palp similar to that of left mandible but article 2 with two inner simple setae, and article 3 with three distal simple setae.

***Labium*** (Fig. 12C) palp covered with setules, with two distal simple setae.

***Maxillule*** (Fig. 12D) outer endite with ten distal spiniform setae and two subdistal setae, inner and outer margins covered with setules; inner endite with one distal trifurcate seta and four distal plumose setae, inner and outer margin covered with setules; palp 2-articled, article 2 with five distal simple setae.

***Maxilla*** (Fig. 12E) outer lobe of movable endite with eight long distal simple setae; inner lobe of movable endite with ~ 12 distal simple setae; outer lobe of fixed endite distal margin with seven blunt, one leaf-like, four trifurcate, two simple setae, and one seta with several spinules on single side; inner lobe of fixed endite distal margin with a row of ~ 25 “articulate” setae (with distinct demarcation on midway of each seta), a cluster of seven simple setae, and four blunt setae with a row of short setules and serrations on distal one third; inner margin with spinules.

***Maxilliped*** (Fig. 12F) basis outer margin covered with setules covered with setules, distal margin with one seta; endite covered with setules, distal margin with five simple setae and six spiniform setae, inner margin with three coupling hooks, inner fold with six plumose setae; palp article 1 outer margin with one distal simple seta, distal margin with three simple setae, article 2 outer margin with seven long distal simple setae, inner margin with five long simple setae and a row of 45–50 shorter simple setae, article 3 outer margin with one distal simple seta, inner margin with ~ 12 simple setae, article 4 with ~ 12 distal simple setae.

***Chelipeds*** (Fig. 13A, B) left and right slightly dimorphic, differences in size and setal pattern. Left cheliped (Fig. 13A) exopod 3-articled, article 3 with four plumose setae; basis 3× as long as broad, dorsal margin with two simple setae on distal half, ventral margin with five long simple setae and four shorter simple setae; merus 0.7× as long as basis, 2.3× as long as broad, covered with totally ~ 25 simple setae; carpus long, ~ 1.9× as long as merus, 4.6× as long as broad, covered with totally ~ 44 simple setae; propodus palm 0.5× as long as carpus, 2.4× as long as broad, with three dorsal simple setae, one outer simple seta near insertion of dactylus, and two inner simple setae, fixed finger 0.8× as long as palm, ~ 4× as long as broad, with six ventral and two short distal simple setae; dactylus plus unguis 6.5× as long as broad, slightly curved. Right cheliped (Fig. 13B) slenderer and less setose than left cheliped, ~ 0.7× as long as left cheliped; exopod 3-articled, article 3 small and naked; basis ~ 3× as long as broad, ventral margin with one subdistal simple seta; merus 0.7× as long as basis, ~ 3× as long as broad, with two outer and two ventral setae; carpus long and slender, 1.9× as long as merus, ~ 5× as long as broad, with one outer subdistal simple seta, dorsal margin with one subdistal simple seta, ventral margin with three midway and one subdistal simple seta; propodus palm 0.4× as long as carpus, 2.1× as long as broad, with one outer simple seta near insertion of dactylus, dorsal margin with one subdistal simple seta, fixed finger 0.7× as long as palm, 3× as long as broad, with two ventral and two distal simple setae, incisive margin with one proximal simple seta; dactylus plus unguis 4.9× as long as broad, slightly curved, with seven subdistal simple setae.

***Pereopod 1*** (Fig. 13C) exopod 3-articled, article 3 with six plumose setae; basis 2.7× as long as broad, dorsal margin with three short, two longer simple setae and a few setules, ventral margin with six simple setae midway and a cluster of eight distal simple setae; ischium with four ventrodistal simple setae; merus 0.6× as long as basis, 1.4× as long as broad, dorsal margin with a cluster of seven distal simple setae and one spiniform seta, ventral margin with a cluster of four simple setae midway, eight simple setae along distal half, three distal simple setae, and one distal short but strong spiniform seta; carpus 0.9× as long as merus, 1.3× as long as broad, distal margin with one long dorsal spiniform seta, a cluster of *ca.* eight dorsal simple setae and two outer setae, ventral margin with nine simple setae and two short but strong spiniform setae; propodus 1.2× as long as carpus, 1.6× as long as broad, dorsal margin with three shorter simple setae on proximal half, six long simple setae and three long spiniform setae on distal half, ventral margin with 11 simple setae and four strong spiniform setae, distal margin with one short subventral simple seta; dactylus (Fig. 13D) plus unguis 0.7× as long as propodus, 5.7× as long as broad, ventral margin with six pointed denticles and one small distal spiniform seta, spiniform seta much shorter than unguis, together subchela-like.

***Pereopod 2*** (Fig. 13E) basis 3.5× as long as broad, with two short dorsal simple setae, one dorsal broom seta, three subventral distal simple setae, and a cluster of five ventrodistal simple setae; ischium with one dorsal simple seta and a cluster of six ventrodistal simple setae; merus 0.4× as long as basis, 1.7× as long as broad, with one outer subdistal and three outer distal simple setae, ventral margin with seven simple setae and one long spiniform seta on distal half; carpus 1.2× as long as merus, 2.2× as long as broad, outer surface with a row of ten simple setae and two long spiniform setae from midway to dorsodistal corner, ventral margin with eight simple setae and two long spiniform setae on distal half, distal margin with three inner simple setae; propodus 0.9× as long as carpus, ~ 3× as long as broad, distal half with a row of five subdorsal simple setae and three spiniform setae, ventral margin with seven simple setae and three spiniform setae; dactylus (Fig. 13F) plus unguis 0.8× as long as propodus, ~ 6× as long as broad, dactylus slightly curved, with one indistinct demarcation midway and one pointed ventrodistal prolongation, unguis curved, slightly longer than prolongation, together chela-like.

***Pereopod 3*** (Fig. 13G) basis 3.2× as long as broad, with a cluster of six ventrodistal simple setae; ischium with a cluster of seven ventral subdistal simple setae; merus 0.3× as long as basis, 1.8× as long as broad, with eight ventral or subventral simple setae and one ventral spiniform seta on distal half, ventral margin slightly denticulate; carpus 1.4× as long as merus, 2.6× as long as broad, ventral to distal margin and subventral surface covered with ~ 16 simple setae and three spiniform setae, ventral margin slightly denticulate; propodus 0.7× as long as carpus, 2.7× as long as broad, with one dorsoproximal broom seta and a row of six simple setae and three spiniform setae from outer midway surface to dorsodistal corner, ventral margin with four simple setae and three spiniform setae on distal half; dactylus (Fig. 13H) attached to propodus subdistally, similar to that of pereopod 2 but not articled.

***Pereopod 4*** (Fig. 14A, figure and description based on non-ovigerous female paratype MBM287305, 7.1 mm long) basis 3.2× as long as broad, with a cluster of five ventrodistal simple setae; ischium with a cluster of six ventrodistal simple setae; merus 0.3× as long as basis, 1.6× as long as broad, with one dorsodistal, two outer subdistal, and three ventral simple setae, ventral margin slightly denticulate; carpus 1.8× as long as merus, 2.5× as long as broad, ventral to distal margin covered with ~ 17 simple setae and six spiniform setae, ventral margin slightly denticulate; propodus 0.6× as long as carpus, 3.2× as long as broad, ventral margin with *ca.* eight simple setae and 10 spiniform setae; dactylus (Fig. 13B, figure and description based on MBM287305) attached to propodus subdistally, nearly as long as propodus, > 9× as long as broad, with three ventral spinules and one long ventrodistal prolongation that is 2.3× as long as unguis, together subchela-like.

***Pereopod 5*** (Fig. 14C) basis 3.2×as long as broad, dorsal margin with two broom setae midway, ventral margin with a cluster of five distal simple setae; ischium dorsal margin with one short subdistal simple seta, ventral margin with a cluster of five distal simple setae, merus 0.3× as long as basis, 1.8× as long as broad, with one dorsodistal simple seta, ventral margin denticulate, six simple setae on distal half; carpus 1.7× as long as merus, ~ 3× as as long as broad, ventral to distal margin covered with ~ 17 simple setae and eight spiniform setae, the distal two spiniform setae very long, ventral margin denticulate; propodus 0.8× as long as carpus, 3.2× as long as broad, ventral margin denticulate, covered with *ca.* ten simple setae and nine spiniform setae; dactylus similar to that of pereopod 3.

***Pereopod 6*** (Fig. 14D) basis long, 4.8× as long as broad, dorsal margin with one subproximal broom seta, ventral margin with two subdistal simple setae; ischium with two dorsal and three ventral subdistal simple setae; merus 0.3× as long as basis, nearly 2× as long as broad, with one dorsodistal simple seta and one simple seta on inner surface, ventral margin with six simple setae on distal half; carpus 1.7× as long as merus, 3.3× as long as broad, with three dorsodistal simple setae, ventral to distal margin with ~ 17 simple setae and five long spiniform setae, ventral margin slightly denticulate; propodus 0.8× as long as carpus, 3.6× as long as broad, with one dorsodistal, one ventrodistal simple seta, two long and one shorter dorsodistal spiniform setae, ventral margin with one proximal simple seta and a row of ~ 28 serrate setae along ventral margin to distal margin; dactylus similar to that of pereopod 5.

***Pleopod 1*** (Fig. 14E, all setae plumose, setules omitted in figure) basal article with seven outer and five inner plumose setae; exopod with 30 plumose setae; endopod with 27 plumose setae, most proximal one on inner margin strong.

***Uropod*** basal article with four or five distal and subdistal simple setae; exopod and endopod not examined.

**Male (allotype MBM287302). *Body*** (Fig. 11H) elongate, dorsoventrally flattened, allotype 5.0 mm long, 6.4× as long as broad. ***Carapace*** subrectangular, ~ 0.1× as long as total body length, 0.8× as long as broad; rostrum rounded, anterior margin flat, with two short simple setae; lateral margin with one simple seta. ***Pereon*** half as long as total body length; each pereonite with at most seven anterolateral simple setae and without or with one posterolateral simple seta, pereonite 1 broadest, as wide as carapace, half as long as broad, pereonite 2 shortest, pereonite 4 longest but narrowest. ***Pleon*** 0.3× as long as total body length, posteriorly narrower; each pleonite similar in length, with several lateral plumose setae, pleonites 1–4 trapezoidal, pleonite 5 subrectangular. ***Pleotelson*** subrectangular, 1.3× as long as pleonite 5, 1.5× as long as broad, with three lateral plumose setae, posterior margin with two pairs of blunt apophyses.

***Antennule*** (Fig. 15A, most aesthetascs represented by fine lines) peduncle article 1 ~ 3× as long as broad, outer margin with 11 simple setae, inner margin with seven simple setae and one strong spiniform seta midway; article 2 ~ 0.5× as long as article 1, 1.6× as long as broad, with one ventral simple seta, outer margin with six simple setae, inner margin with one simple seta on proximal half and a cluster of three subdistal simple setae; article 3 short, 0.4× as long as article 2, 0.7× as long as broad, distal margin with three outer and two inner simple setae; article 4 with one inner simple seta; outer flagellum 21-articled, *ca.* as long as peduncle, proximal articles shorter and broader, covered with numerous aesthetascs, distal articles longer and slenderer, each article without or with at most four distal simple setae; inner flagellum 12-articled, slightly longer than outer flagellum, articles 5 and 7 with one distal broom seta, each article without or with at most four distal simple setae.

***Antenna*** (Fig. 15B, most aesthetascs represented by fine lines) peduncle article 1 naked; article 2 with two short outer simple setae, squama elongate, with three outer, three inner, two longer and one short distal simple seta; article 3 very short, naked; article 4 longest, 0.8× as long as broad, with three short outer simple setae; article 5 similar to flagellum articles, covered with aesthetascs, with one outer distal simple seta; flagellum 20-articled, each article very short, proximal articles broader, outer half covered with numerous aesthetascs, each article without or with at most four distal simple setae.

***Left cheliped*** (Fig. 15C, figure and description based on male paratype MBM287307, 4.2 mm long) basis proximal part missing, dorsal margin with one short subdistal simple seta, ventral margin with one short and two longer simple setae; merus 2.2× as long as broad, outer surface with one subdistal simple seta, inner surface with two dorsodistal simple setae, ventral margin with four subdistal simple setae; carpus 2.1× as long as merus, 3.5× as long as broad, with seven ventral simple setae, inner surface with *ca.* ten subdorsal simple setae; propodus palm and fixed finger combined ~ 0.8× as long as carpus, palm 1.8× as long as broad, with one long outer simple seta near insertion of dactylus, dorsal margin with one subproximal and three subdistal simple setae, inner surface with two long subdistal simple setae, fixed finger 2.7× as long as broad, with six ventral, one outer and one inner simple seta, incisive margin with one large and blunt apophysis, and six distal simple setae; dactylus curved, 4.7× as long as broad. *Right cheliped* (Fig. 15D) exopod 3-articled, article 3 with four plumose setae; basis 2.3× as long as broad, ventral margin with one longer and three short but strong simple setae on proximal half, and one longer simple seta on distal half; merus 0.6× as long as basis, 2.4× as long as broad, ventral margin with four simple setae on proximal half, four longer and one short but strong simple setae on distal half, distal margin with two outer and three inner simple setae; carpus 1.7× as long as merus, 3.4× as long as broad, with one inner subventral simple setae, ventral margin with five simple setae and three subdistal simple setae; propodus attached to carpus subdistally, palm half as long as carpus, almost 2× as long as broad, with one outer simple seta near insertion of dactylus, dorsal margin with one proximal simple seta, a cluster of three simple setae on proximal half, one midway and a row of five subdistal simple setae, fixed finger distal part missing, with six ventral and three distal simple setae, incisive margin crenulate; dactylus plus unguis as long as palm, with three outer simple setae, unguis long and slender.

##### Remarks.

The specimens of *Swireapseudesplanafrontis* sp. nov. are extremely fragile, with appendages frequently missing. This phenomenon was also found in species of some other parapseudid genera, e.g., *Parapseudes*, *Pakistanapseudes* and *Saltipedis*, and considered to be autotomy (Guţu 1996). As a result, the morphological description of *S.planafrontis* could only be made up by the sum of parts of the several paratypes and allotype. It may be a novel discovery that the chelipeds of the present species not only display sexual dimorphism but also exhibit a little dimorphism between left and right chelipeds of a single female individual. Unfortunately, no male specimens were collected with complete left and right chelipeds, so it is not possible to confirm that the morphological differences between right cheliped of the allotype and left cheliped of the male paratype MBM287307 are the results of dimorphism or simply the result of variation (Figs 13A, B, 15C, D).

Apart from the dimorphism of female left and right cheliped, there are several other unique morphological features that easily distinguish *Swireapseudesplanafrontis* sp. nov. from the two previously recorded species, *S.birdi* and *S.toloensis*: 1) rostrum distally wide and flat; 2) the presence of one strong spiniform seta midway on the inner margin of the antennule peduncle article 1; 3) the presence of one inner distal spiniform seta on the female antenna article 2; 4) the presence of one small ventrodistal spiniform seta (rather than prolongation) on pereopod 1 dactylus; 5) the long ventrodistal prolongation on pereopod 4 dactylus (Figs 11A–C, H, 13C, D, 14A, B, 15A, Table 3; see also Guţu and Iliffe 2008: figs 1A–D, 2D, 3C, 4A; Bamber 1997: figs 7A, B, 8A, B, 9H).

**Table 3.** Morphological comparison among all species of *Swireapseudes*.

Character / Species name	*S.planafrontis* sp. nov.	*S.birdi*	*S.toloensis*
**Carapace**			
Shape of rostrum	wide and rounded, distally flat	rounded, distally pointed	rounded, distally pointed
**Antennule**			
Number of female outer/inner flagellum articles	16/13	14/10	18/12
**Antenna**			
Number of female flagellum articles	14	8	14
**Pereopod 1**			
Shape of merus and carpus ventral spiniform setae	short and strong	long and slender	short and strong
Form of dactylus	subchelate	chelate	subchelate
**Pereopod 4**			
Form of dactylus	ventrodistal prolongation very long	simple, without unguis	subchelate
**Pereopods 2, 3, 5, 6**			
Form of dactylus	chelate	chelate	subchelate
**References**	present study	Guţu and Iliffe 2008	Bamber 1997

#### ﻿Key to the species of *Swireapseudes* (see also Table 3)

**Table:** 

1	Rostrum distally wide and flat	***S.planafrontis* sp. nov.**
–	Rostrum distally pointed	**2**
2	Pereopod 1 merus, carpus, and propodus with short and strong ventral spiniform setae	***S.toloensis***
–	Pereopod 1 merus, carpus, and propodus with long and slender ventral spiniform setae	***S.birdi***

## Supplementary Material

XML Treatment for Apseudes

XML Treatment for Apseudes spinidigitus

XML Treatment for Phoxokalliapseudes

XML Treatment for Phoxokalliapseudes shandongensis

XML Treatment for Swireapseudes

XML Treatment for Swireapseudes planafrontis
